# Evolution and Structural Organization of the C Proteins of *Paramyxovirinae*

**DOI:** 10.1371/journal.pone.0090003

**Published:** 2014-02-25

**Authors:** Michael K. Lo, Teit Max Søgaard, David G. Karlin

**Affiliations:** 1 Centers for Disease Control and Prevention, Viral Special Pathogens Branch, Atlanta, Georgia, United States of America; 2 Division of Structural Biology, Oxford University, Oxford, United Kingdom; 3 Department of Zoology, University of Oxford, Oxford, United Kingdom; Institute of Infectious Disease and Molecular Medicine, South Africa

## Abstract

The phosphoprotein (P) gene of most *Paramyxovirinae* encodes several proteins in overlapping frames: P and V, which share a common N-terminus (PNT), and C, which overlaps PNT. Overlapping genes are of particular interest because they encode proteins originated *de novo*, some of which have unknown structural folds, challenging the notion that nature utilizes only a limited, well-mapped area of fold space. The C proteins cluster in three groups, comprising measles, Nipah, and Sendai virus. We predicted that all C proteins have a similar organization: a variable, disordered N-terminus and a conserved, α-helical C-terminus. We confirmed this predicted organization by biophysically characterizing recombinant C proteins from Tupaia paramyxovirus (measles group) and human parainfluenza virus 1 (Sendai group). We also found that the C of the measles and Nipah groups have statistically significant sequence similarity, indicating a common origin. Although the C of the Sendai group lack sequence similarity with them, we speculate that they also have a common origin, given their similar genomic location and structural organization. Since C is dispensable for viral replication, unlike PNT, we hypothesize that C may have originated *de novo* by overprinting PNT in the ancestor of *Paramyxovirinae*. Intriguingly, in measles virus and Nipah virus, PNT encodes STAT1-binding sites that overlap different regions of the C-terminus of C, indicating they have probably originated independently. This arrangement, in which the same genetic region encodes simultaneously a crucial functional motif (a STAT1-binding site) and a highly constrained region (the C-terminus of C), seems paradoxical, since it should severely reduce the ability of the virus to adapt. The fact that it originated twice suggests that it must be balanced by an evolutionary advantage, perhaps from reducing the size of the genetic region vulnerable to mutations.

## Introduction

**Figure 1:**
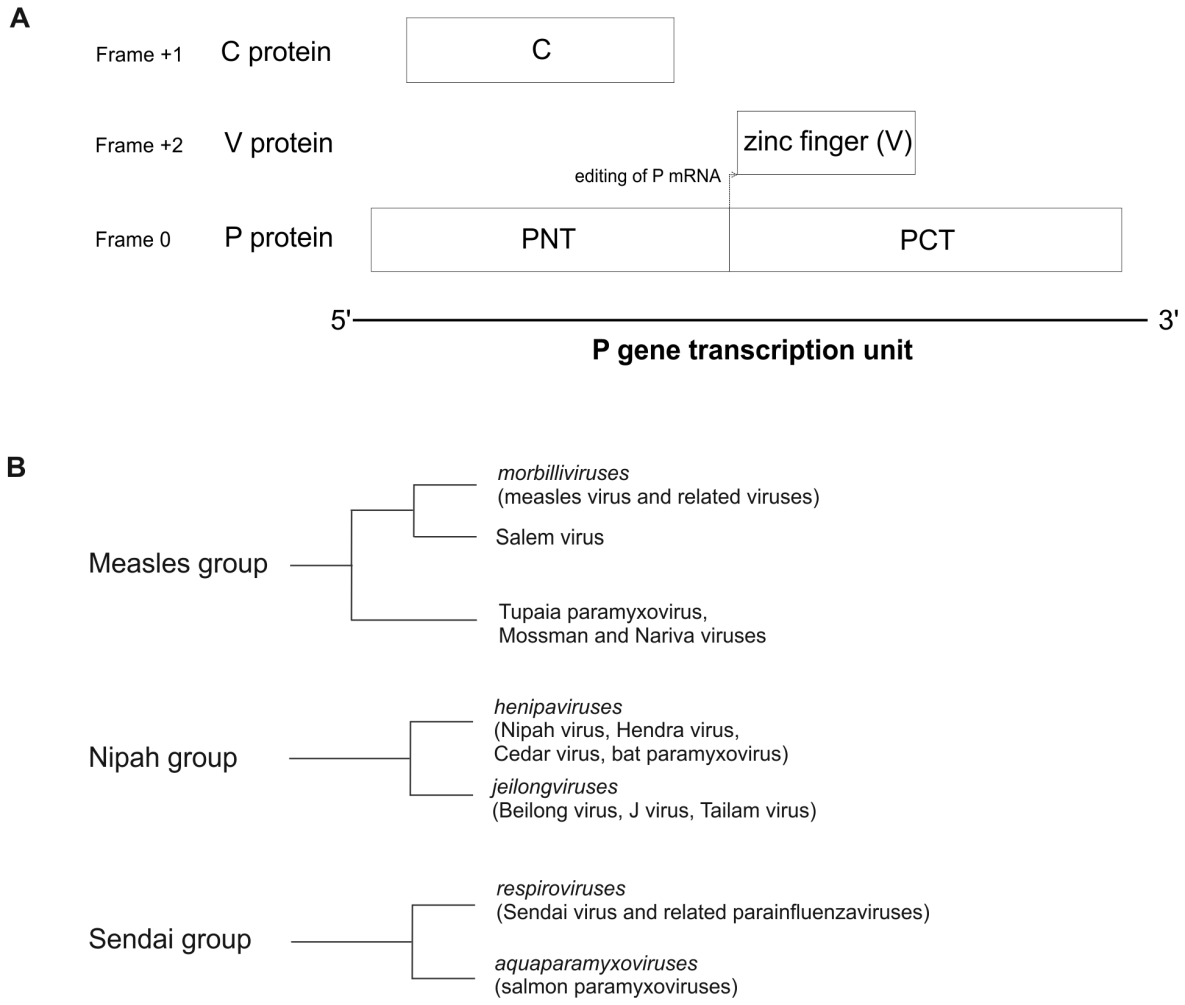
Organization of the P/V/C gene of *Paramyxovirinae* and phylogeny of the C proteins. A. Organization of the P/V/C gene transcription unit of *Paramyxovirinae*. PNT: N-terminal moiety of P; PCT: C-terminal moiety of P. The V protein is composed of PNT fused, by co-transcriptional editing (arrow) of the P mRNA, to a zinc finger domain encoded in a different frame. For clarity, only the C-terminal zinc finger of V is shown. B. Clustering of *Paramyxovirinae* C proteins by sequence similarity. The cladograms represent the measles, Nipah and Sendai groups.

*Paramyxovirinae* is a large virus subfamily that contains 9 known human pathogens: measles virus, mumps virus, human parainfluenza viruses type 1 (hPIV1), 2, 3 and 4, Menangle virus, and the recently emerged, highly pathogenic Nipah and Hendra viruses [1]. *Paramyxovirinae* encode multiple proteins from the phosphoprotein (P) gene transcription unit, including P, V, and C. In almost all *Paramyxovirinae*, the P gene mRNA is edited, resulting in the expression of at least two proteins, P and V, which share an identical N-terminus (PNT), but have a unique C-terminus (Figure 1A) (for a review, see [2]). In addition, several genera, including *Morbilliviruses*, *Henipaviruses*, and *Respiroviruses*, encode a third protein, C, within their P gene, from an overlapping reading frame [2]. The C proteins are expressed by a variety of mechanisms including: leaky scanning [3]–[5], non-AUG start codons [6], [7], ribosomal shunting [8], and proteolytic processing [9]. The region of P that overlaps C, corresponding approximately to PNT (Figure 1A), is disordered [10]–[13], and contains conserved sequence motifs, such as soyuz1, found in all *Paramyxovirinae,* which binds the viral nucleoprotein, and soyuz2, of unknown function [14].

The two primary functions of the C proteins are their abilities to regulate viral transcription/replication and to antagonize the antiviral responses of the host. These functions are thought to be interconnected, since a decrease in viral transcription/replication often correlates with a decrease in the innate antiviral responses of the host [15]–[18] (for a review, see [19]). Most paramyxoviral C proteins inhibit viral RNA synthesis, and thereby presumably regulate viral gene expression [20]–[24]. However, they differ in the degree to which they block host antiviral responses [25]. These responses are composed of two crucial signaling cascades: A) *Induction* of type I interferon (IFN), following *recognition* of virus-derived elements by pattern recognition receptors (PRRs) and B) IFN *signaling* through the JAK/STAT pathway, leading to transcription of antiviral effector genes [26], [27].

Most paramyxoviral C proteins can inhibit IFN induction, but only *respiroviruses* are known to inhibit IFN signaling. *Morbillivirus* C proteins have two mechanisms to counteract IFN induction: 1) by reducing levels of viral replication, which limits the production of viral patterns recognized by PRRs and prevents them from inducing IFN [17], [21], [28]; and 2) by inhibiting IFN transcription in the nucleus [29], [30]. An initial study reported that *measles virus* C protein blocks IFN signaling [31], but subsequent studies indicated that this effect is not significant [17], [32], [33]. Similarly, although the mechanistic details are less clear, *Henipavirus* C proteins block IFN induction by decreasing viral RNA synthesis, which indirectly inhibits type I IFN induction; but they have minimal effects on IFN signaling [15], [34]–[37]. Like the *morbilliviruses*, *Respirovirus* C proteins also counteract IFN induction through two mechanisms: 1) by minimizing production of double-stranded RNA (dsRNA), thereby avoiding PRR activation [16], [38]; and 2) by inhibiting IRF3-dependent induction of type I IFN [39]. However, the C proteins of *respiroviruses* differ from those of *Morbilliviruses* and *Henipaviruses* in being also able to inhibit IFN signaling [16], [26], [38]–[53]. Finally, a new role has been reported recently for the C proteins of *respiroviruses*: they regulate the levels of viral genomes and antigenomes produced during infection [54].

Interestingly, *henipaviruses* and *morbilliviruses* can also block IFN signaling, but do so by proteins encoded by the P frame rather than the C frame (i.e. P, V, or a third protein called W), which interfere with the localization or phosphorylation of STAT1 (Signal Transduction Activator of Transcription 1), among other mechanisms [55]–[62].

Overlapping genes, such as those encoding P and C, are of particular interest because they encode proteins originated *de novo* (in contrast to origination by well-characterized processes such as gene duplication or horizontal gene transfer [63], [64]). Indeed, overlapping genes are thought to arise by overprinting, a process in which mutations within an existing (“ancestral”) protein-coding reading frame allow the expression of a second reading frame (the *de novo* frame), while preserving the expression of the first frame [65]–[67]. *De novo* proteins have been little studied but are known to play an important role in viral pathogenicity [68], [69], for instance by neutralizing the host interferon response [70] or the RNA interference pathway [71]. In addition, *de novo* proteins characterised so far have previously unknown 3D structural folds [68], [71], [72] and novel mechanisms of action [71]. Thus, this class of proteins may challenge the notion that nature only utilizes a limited number of different protein folds and that this fold space is well mapped [73], [74]. Another particularly interesting feature of overlapping genes is the evolutionary paradox they present, since the overlap imposes sequence constraints which should restrict the ability of the virus to adapt [75]–[81].

Our study was divided in three strands. First, we predicted the structural organization of the C proteins, and determined whether they had detectable sequence similarity, which could indicate a common origin, guide experimental studies, and facilitate 3D structure determination [82]. Second, we verified our predictions experimentally, by expressing, purifying and characterizing several C proteins in bacteria. Third, we investigated the evolutionary history of the P/C gene overlap, and tried to determine which, of P and C, is the novel frame.

## Methods

### Sequence Alignment

**Figure 2:**
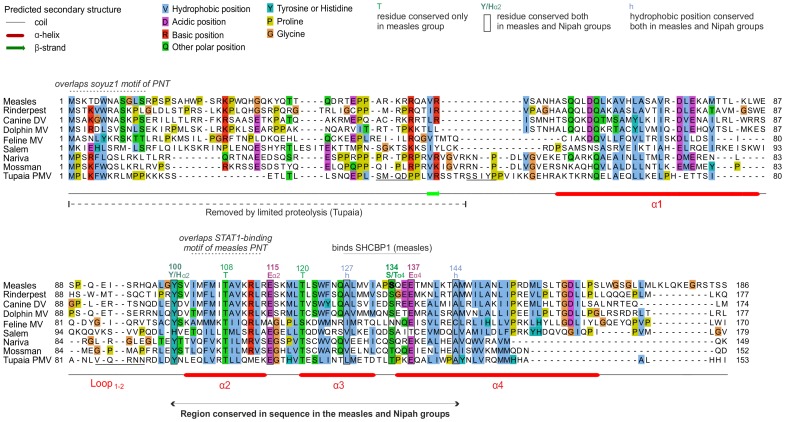
Alignment of the C proteins of the measles group. Numbering corresponds to *measles virus*. The N-terminus of C is highly variable and shown for information only. Only the C-terminal moiety of C (helices α2 to α4) is reliably aligned; positions that appear conserved but are outside this region are thus not indicated. Residues that have been experimentally substituted (Table 2) are in bold. N-terminal sequences of fragments of Tupaia PMV C obtained after limited proteolysis are underlined. Overlapping motifs of the PNT frame overlapping C are indicated above the alignment.

The accession numbers of the sequences of *Paramyxovirinae* P used in this study, as well as the abbreviations of species names, are in Table 1. The sequence of the C protein of *Pacific salmon paramyxovirus* [83], [84] was generously made available by Bill Batts and Jim Winton. We used Psi-Coffee [85], [86] for multiple sequence alignments (MSAs). All alignments are presented using Jalview [87] with the ClustalX colouring scheme (see Figure 2b and 2d in [88]). The aligned sequences of the C proteins in text format are in File S1.

**Table 1:** Accession numbers of *Paramyxovirinae* C proteins.

Virus species	Abbreviation	Genus	Genbank accession number
*Atlantic salmon paramyxovirus*	Atlantic PMV	Unclassified	ABW38050.1
*Beilong virus*	Beilong	Jeilongvirus*	YP_512248.1
*Bovine parainfluenza virus 3*	bPIV3	*Respirovirus*	P06164
*Canine distemper virus*	Canine DV	*Morbillivirus*	NP_047203.1
*Dolphin Morbillivirus*	Dolphin MV	*Morbillivirus*	NP_945026.1
*Bat paramyxovirus/Eid_hel/GH-M74a/GHA/2009*	Bat PMV	*Henipavirus*	AFH96008.1
*Cedar virus*	Cedar	*Henipavirus*	AFP87276.1
*Feline morbillivirus*	Feline MV	*Morbillivirus*	AFH55514.1
*Hendra virus*	Hendra	*Henipavirus*	O55779
*Human parainfluenza virus 1*	hPIV1	*Respirovirus*	NP_604434.1
*Human parainfluenza virus 3*	hPIV3	*Respirovirus*	NP_599251.1
*J virus*	J virus	Jeilongvirus*	YP_338079.1
*Measles virus*	Measles	*Morbillivirus*	NP_056920.1
*Mossman virus*	Mossman	Unclassified	NP_958051.1
*Nariva virus*	Nariva	Unclassified	YP_006347585.1
*Nipah virus*	Nipah	*Henipavirus*	AAY43913.1
*Pacific salmon paramyxovirus*	Pacific PMV	Unclassified	AFF60402.1
*Peste des petits ruminants virus*	PPRV	*Morbillivirus*	YP_133824.1
*Phocine distemper virus*	Phocine DV	*Morbillivirus*	P35940.1
*Porcine parainfluenza virus 1*	pPIV1	*Respirovirus*	AGR39559.1
*Rinderpest virus*	Rinderpest	*Morbillivirus*	ADF32062.1
*Salem virus*	Salem	*Morbillivirus*-like	Q9IZB9
*Sendai virus*	Sendai	*Respirovirus*	NP_056872.1
*Tailam virus*	Tailam	Jeilongvirus*	AEU08859.1
*Tupaia paramyxovirus*	Tupaia PMV	Unclassified	Q9WS38

(*) Proposed genus.

We used two criteria to estimate the reliability of alignments of the C proteins: 1) the CORE reliability index, which is based on the agreement between the different alignment programs used by Psi-Coffee, and is part of the standard output of Psi-coffee [86]; 2) in the case of the measles and Nipah groups, we also considered the coherence between the alignments of either group separately and the alignment of both groups. We considered as not reliably aligned the positions that either have a low Psi-coffee CORE index, or are not aligned in the same way in these alignments.

Finally, we used TranslatorX [89] to generate a nucleotide alignment of the P/C gene corresponding to an amino acid alignment of the C protein. The alignment of the C proteins (not shown) was created using the MUSCLE program [90] built in TranslatorX, and is thus slightly different from that generated by Psi-coffee, mainly in the region between E_α2_ and S/T_α4_. This has no impact on the results presented.

### Sequence Analyses

The secondary structure of individual sequences was predicted using Jpred [91], and was verified in the context of multiple alignments using PROMALS [92]. We predicted disordered regions with MetaPrDOS [93], according to the principles described in [94]. We used HHalign [95] to compare the MSAs of the C proteins of various groups, with a cutoff E-value of 10^−5^.

To identify and cluster homologous C proteins, we performed iterative sequence searches [96] on the C proteins of each taxon, using csi-blast [97] and HHblits [98] with a cutoff E-value of 10^−3^, as described in [99]. We identified 5 subgroups of homologs (Figure 1), formed by the following taxons: 1) the genus *morbillivirus* and *Salem virus*; 2) *Tupaia Paramyxovirus, Mossman virus*, and *Nariva virus;* 3) the genus *henipavirus;* 4) the newly proposed genus *jeilongvirus*; and 5) the two genera *respirovirus* and *aquaparamyxovirus* (called “Sendai group”). Several proteins of subgroups 1 and 3 had a subsignificant (E>10^−3^) similarity with proteins of subgroups 2 and 4, respectively, indicating that these subgroups may be homologous [99]. We confirmed their homology by using HHalign [95] (E = 5.10^−11^ for the comparison between subgroups 1 and 2, and E = 2.10^−9^ for the comparison between subgroups 3 and 4). We called the combination of subgroups 1 and 2 “measles group” and the combination of subgroups 3 and 4 “Nipah group”.

### Cloning of the C Genes

To maximize our chances of successfully expressing C proteins, we adopted a high-throughput approach. We cloned full-length synthetic cDNAs (obtained from Genscript) of the C proteins of all 24 species in the measles, Nipah and Sendai groups into the vector pOPIN-F [100] using the InFusion procedure, as described in [100], [101]. The resulting fusion proteins have an N-terminal hexahistidine tag followed by a 3C cleavage site immediately upstream of the coding sequence of the C proteins.

### Expression of the C Proteins

Proteins were expressed in the bacteria *Escherichia coli* (*E. coli*) using the BL21(DE3) Rosetta pLysS strain (Novagen), following the ZYM-5052 auto-induction protocol [102]. Briefly, large scale cultures were inoculated to OD600 of 0.02 and grown for 16 h at 25°C. Cells were harvested and the pellet resuspended 1∶3 (w/vol) in lysis buffer (50 mM TrisHCl, 500 mM NaCl, 30 mM Imidazole pH 8.0, 1% vol/vol Protease inhibitor mix (Sigma P8849)) and frozen in liquid nitrogen before storage at −80°C.

### Purification of the C Proteins

We purified both C proteins in two steps: Nickel Immobilized Affinity Chromatography (IMAC) followed by size-exclusion chromatography (SEC). Pellets were thawed and homogenized (Constant Systems homogenizer) at 25 kpi at 4°C. The lysate was cleared at 50,000 g for 30 minutes before batch incubation of the supernatant (i.e. the soluble fraction of bacteria) on Ni-NTA sepharose FF resin (Qiagen) for 2 hrs at 4°C. The material was collected in an Econo-Pac column (Biorad) and washed in 100 Column Volumes (CV) of lysis buffer. Elution was done in 0.5 CV fractions with lysis buffer containing 500 mM Imidazole. Fractions containing protein were pooled and loaded onto a preparative Superdex 75 (GE Healthcare Life Sciences) size exclusion column pre-equilibrated in 20 mM Tris 150 mM NaCl, 1 mM EDTA, pH 7.5. Peak fractions were pooled and concentrated using 15 ml spin concentrators (Millipore).

### Circular Dichroism (CD)

Protein samples were extensively dialyzed into 20 mM NaPhosphate, 20 mM NaCl pH7.5 and then concentrated to 0.2 mg/ml in spin concentrators (0.5 ml, 3KDa MWCO, Millipore). The Circular dichroism (CD) analysis was done on a JASCO 815 CD spectropolarimeter. Data are averages of 5 independent scans in the 190 nm –250 nm range, and were normalized to the baseline of the dialysis buffer. The data were smoothed using the manufacturer’s software (Jasco SpectraManager) before interpretation. The percentage of α-helix was calculated according to the formula: percentage of α-helix = (θ_208–_4000)/(-33000-4000)×100, where θ_208_ is the ellipticity at 208 nm [103].

### Limited Proteolysis

From 1 mg/ml protease stocks, we made 10-fold serial dilutions in 20 mM Hepes, 50 mM NaCl, 10 mM MgSO4, pH 7.5. Proteins were concentrated to 0.6 mg/ml by spin concentrators (0.5 ml, 3 MWCO, Millipore). For limited proteolysis, 10 µl of protein was mixed with 3 µl of protease and incubated on ice for 30 min, 60 min or 2 hrs. Reactions were stopped by adding 2 µl protease inhibitor mix (Sigma P8849). To each reaction, 5 µl of 4x SDS PAGE sample buffer was added and samples were heated to 95°C for 2 min before loading on a 1 mm 15% SDS-PAGE gel. A subtilisin digest of hPIV1 C and an α-chymotrypsin digest of Tupaia PMV C gave rise to stable fragments which were blotted to PVDF before submitting the samples for N-terminal sequencing (ALTA bioscience, UK).

### Analytical Size Exclusion Chromatography (SEC)

Analytical size exclusion chromatography (SEC) was performed at a flow-rate of 0.5 ml/min using a Superdex 75 10/300 column (GE Healthcare Life Sciences) pre-equilibrated in 20 mM TrisCl, 150 mM NaCl, 1 mM EDTA pH = 7.9. The column was calibrated with a separate run of appropriate globular marker proteins (Gel Filtration LMW Calibration Kit, GE Healthcare Life Sciences).

## Results

### The C Proteins of Paramyxovirinae Cluster in three Groups: the Measles, Nipah and Sendai Groups

On the basis of sequence analyses (see Methods), the C proteins of *Paramyxovirinae* can be divided into three groups: the measles, Nipah and Sendai groups (Figure 1B). The measles group is composed of *morbilliviruses*, of the unclassified *Salem virus*, and of a subgroup comprising the unclassified *Tupaia paramyxovirus, Mossman virus* and *Nariva virus*. The Nipah group comprises *henipaviruses* and *jeilongviruses*. Finally, the Sendai group is composed of *respiroviruses* and of the recently described genus *aquaparamyxovirus*, composed of fish viruses [83], [84], [104] related to *respiroviruses* [105], [106]. The classification of C into measles and Nipah groups is supported by an examination of the PNT domain of P, which is encoded by the same region as C but in a different frame (Figure 1A). Indeed, the PNT of all species in the Nipah group differ from the PNT of the measles group in having a soyuz2 motif (see Introduction) [14].

We found that other *Paramyxovirinae* that do not not express a C frame [2], [107] can be classified in two groups based on the phylogeny of their P gene: the mumps group (comprised of the sister genera *rubulavirus* and *avulavirus*) and the Fer de lance group (formed by the genus *ferlavirus* [108]). This classification corresponds to that of previous analyses [105].

### The C Proteins of the Measles and Nipah Groups are Homologous

**Figure 3:**
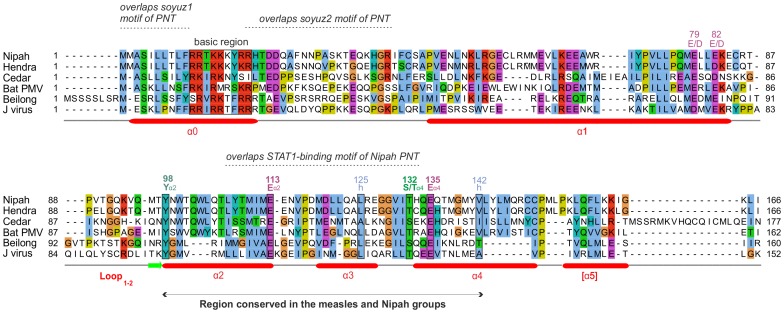
Alignment of the C proteins of the Nipah group. Conventions are the same as in Figure 2. Numbering corresponds to *Nipah virus*. Several residues that appear conserved have not been indicated, because their alignment is not reliable, or their conservation is probably imposed by the P frame (see text).

**Figure 4:**
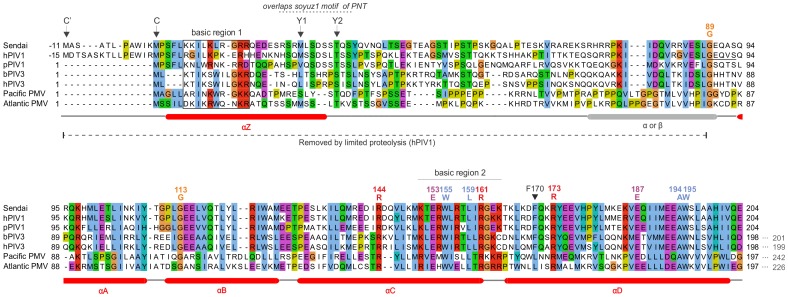
Alignment of the C proteins of the Sendai group. Conventions are the same as in Figure 2. Numbering corresponds to the C protein of *Sendai virus*. Arrows indicate the start of the different isoforms of C. For information, the arrowhead indicates the well-characterized F residue of *respiroviruses* (F170 in *Sendai virus*), whose substitution by S reduces innate immune antagonism and attenuation of *in vivo* pathogenesis by C [39], [53], [153]–[155] (see Table S1). The N-terminal sequence of the fragment of hPIV1 C obtained after limited proteolysis is underlined. The variable region between basic region 1 and residue G89 is not reliably aligned and is presented for information only.

We separately aligned the C proteins of the measles, Nipah and Sendai groups (Figures 2, 3, and 4 respectively; the aligned sequences in text format are in file S1). In these three groups, we observed a similar organization of the C proteins, composed of a variable N-terminus predicted to be disordered, and of a C-terminus predicted to be ordered and α-helical. We compared these alignments to each other using the profile-profile comparison software HHalign [95] (see Methods). Briefly, a sequence profile is a representation of a multiple alignment that contains information about which amino acids (aas) are “tolerated” at each position of the alignment, and with what probability. Comparing profiles is much more sensitive than comparing single sequences, because the profiles contain information about how the sequences can diverge and thus can identify weak similarities which remain after both sequences have diverged [99], [109], [110].

**Figure 5:**
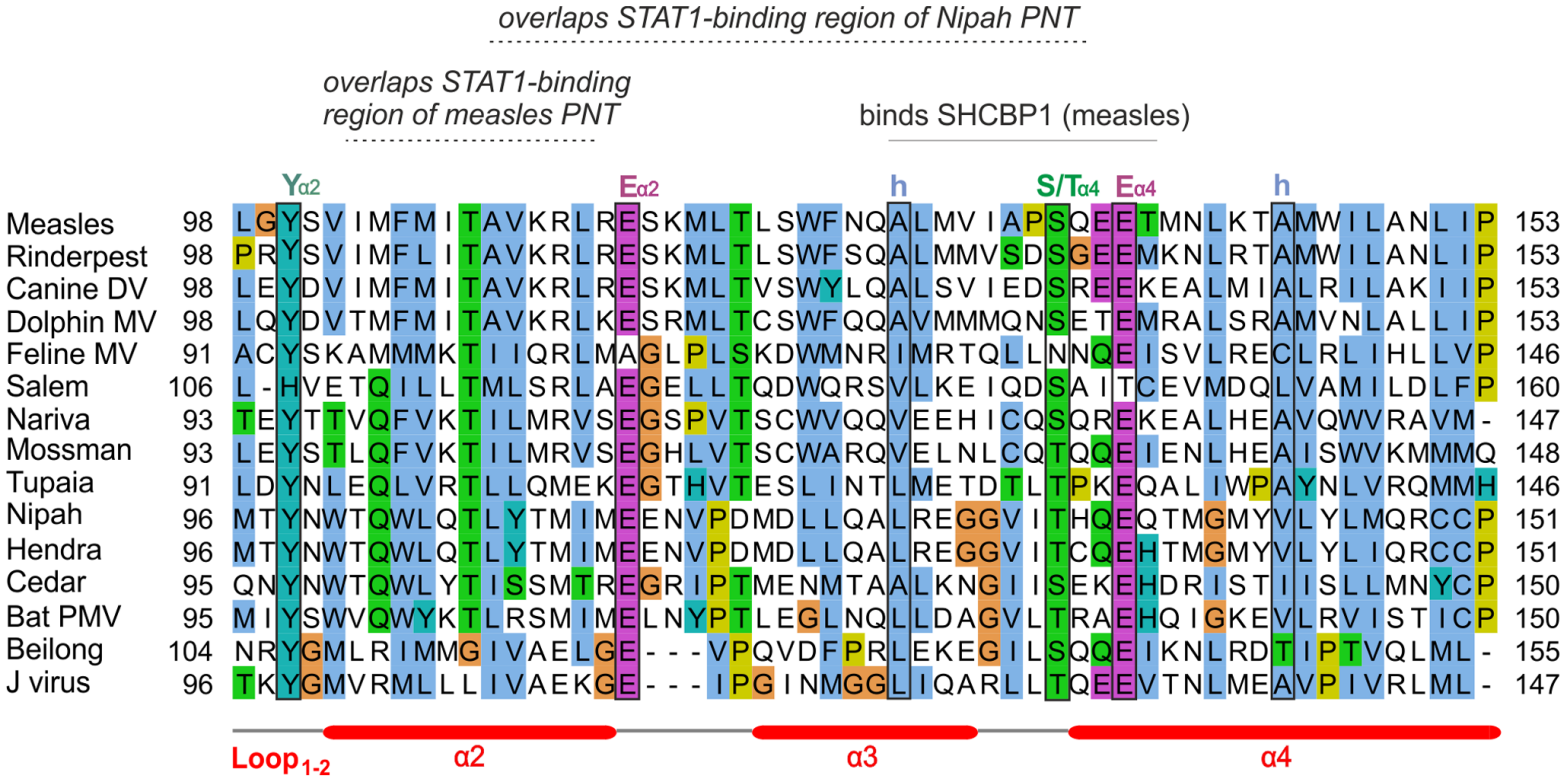
Alignment of the C proteins of the measles and Nipah groups. Conventions are the same as in Figure 2. Several positions appear conserved but have not been indicated, because their alignment is not reliable (see text).

HHalign reported that the C proteins of the measles and Nipah groups have statistically significant similarity (E = 4×10^−6^) over a region of about 50aa in their C-terminus (shown in Figure 5). This high similarity could in theory result either from convergent evolution or from homologous descent. The fact that the measles and Nipah groups are phylogenetically related [105], and that their C proteins are encoded in the same genomic location makes homologous descent a much more likely explanation. On the other hand, HHalign did not detect any similarity between the C proteins of the Sendai group and those of the measles and Nipah groups. Thus, either they are not homologous, despite their similar organization, or they are homologous but have diverged in sequence beyond recognition. The latter scenario is possible, in theory, since the relative frame of C compared to P (+1) is the same in the Sendai group and in the measles/Nipah groups (Figure 1A).

### Sequence Analysis of the C Proteins of the Measles and Nipah Groups

Figures 2 and 3 present alignments of the C proteins of the measles and Nipah groups, respectively. Above the alignments, we indicated regions of C that overlap conserved motifs of the P frame. The C proteins of the measles and Nipah groups are all composed of a 30–60 amino acid (aa) N-terminus predicted to be at least partially disordered, and of a 90–120 aa C-terminus comprising a predicted α-helix (α1), a loop of 10–20aa (“loop_1–2_”), and three further α-helices (α2 to α4), followed in some species by C-terminal extensions of at most 20aa (forming helix α5 in some species of the Nipah group).

In the C proteins of the measles group, only the region from α2 to α4 is well conserved in sequence; it contains many conserved positions (Figure 2), of which six (boxed) are also conserved in the C proteins of the Nipah group (see below). In contrast, the C proteins of the Nipah group contains two additional, conserved regions (Figure 3): 1) a short N-terminus with α-helical potential (α0, aa 2–19 in Nipah virus), containing a hydrophobic region followed by a basic region (boxed in Figure 3); and 2) a short region at the C-terminus of α1 (aa 74–83 in Nipah virus) that contains two conserved acidic positions (E/D). The apparent conservation of other regions of C, which overlap the soyuz1 and soyuz2 motifs of the P frame (Figure 3), should not be over-interpreted, since it may be due to constraints imposed by selection pressures acting in fact on the P frame, which is much more conserved than the C frame in these regions (not shown).

An alignment of the C proteins of both groups (Figure 5) revealed four remarkable positions conserved in nearly all viruses (boxed in Figure 5): a Tyrosine (Y) upstream of helix α2 (Y_α2_); a Glutamate (E_α2_) at the C-terminus of the same helix; a residue with an alcohol group (Serine/Threonine, S/T_α4_) at the N-terminus of helix α4; and a Glutamate (E_α4_) two residues downstream. Two other positions of hydrophobic nature (indicated by “h”) are conserved in both groups. These conserved residues are also boxed in Figures 2 and 3, in the separate alignments of the measles and Nipah groups. Other positions that appear conserved in Figure 5 or in Figures 2 and 3 may in fact not be reliably aligned (see Methods) and are therefore not boxed.

### Sequence Analysis of the C Proteins of the Sendai Group

Figure 4 shows the alignment of the C proteins of the Sendai group. In *Sendai virus* and *human parainfluenza virus 1* (hPIV1), as many as four products (C’, C, Y1, Y2) are expressed from the C reading frame by a combination of alternative initiation codons [6]–[8] and proteolytic processing [9]. Their respective N-termini are indicated by arrows. The C proteins of the Sendai group have a similar organization to that of the measles and Nipah groups. They are composed of a variable, disordered N-terminus of about 80aa, rich in Prolines (P), Serines (S) and Threonines (T), followed by a conserved C-terminus composed of four α-helices (αA to αD). The N-terminus contains a basic region (boxed in Figure 4) within a predicted α-helix (αZ), like the C protein of the Nipah group (Figure 3). In the C protein of *Sendai virus*, the first half of αZ was reported to act as a membrane-targeting signal, perhaps by forming an amphipathic α-helix [111]. There are 11 residues strictly conserved in C across the Sendai group, clustered predominantly in the C-terminus of αC and in αD. αC is particularly rich in K and R (“basic region 2” in Figure 4), suggesting it might bind a negatively charged partner.

### Obtaining a Reliable Alignment of the Region of PNT Containing STAT1-binding Sites in *measles virus* and *Nipah virus*

**Figure 6:**
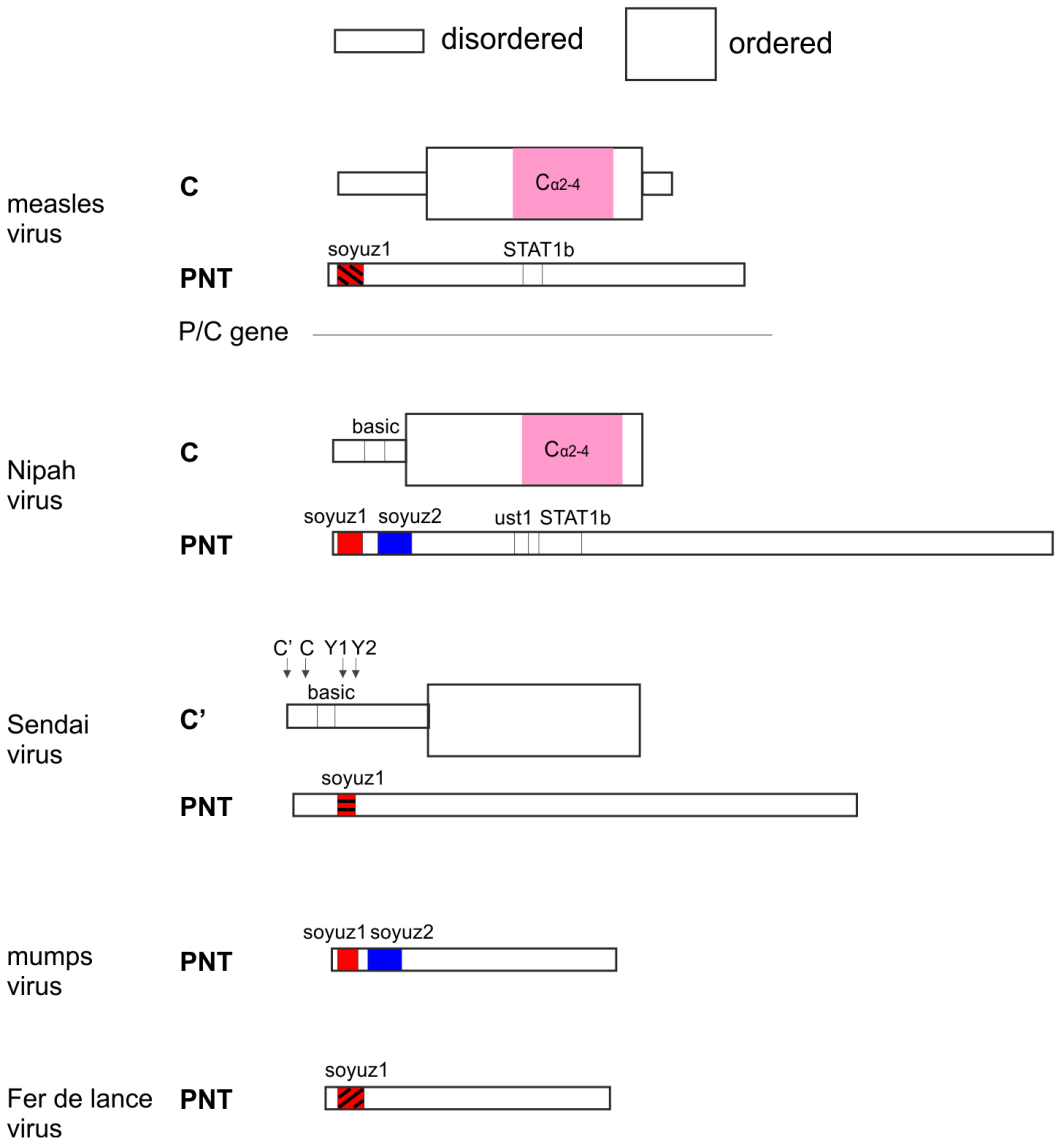
Summary of the organization of *Paramyxovirinae* PNT and C. The figure is to scale, with PNT and C vis-à-vis of each other. The PNTs are all positioned so that their soyuz1 motifs match. Regions whose homology is proven (by statistically significant similarity) have the same color. Homology of soyuz1 motifs is suspected but not proven [14], thus they have a same color, but different patterns. STAT1b: STAT1-binding site. Ust1: “upstream of STAT1” motif.

**Figure 7:**
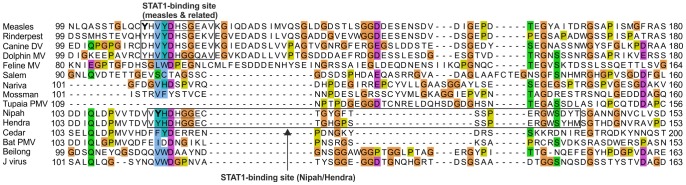
Alignment of the central region of PNT of the measles and Nipah groups (not corrected by using the C frame). Note the high variability of the alignment. The [Y/H]DH[S/G]GE motifs common to the STAT1-binding sites of PNT of *measles virus* and *Nipah virus* are underlined. In bold are the residues Y110 of *measles virus* PNT and Y116 of *Nipah virus* PNT, which were suggested to be analogous (see text).

**Figure 8:**
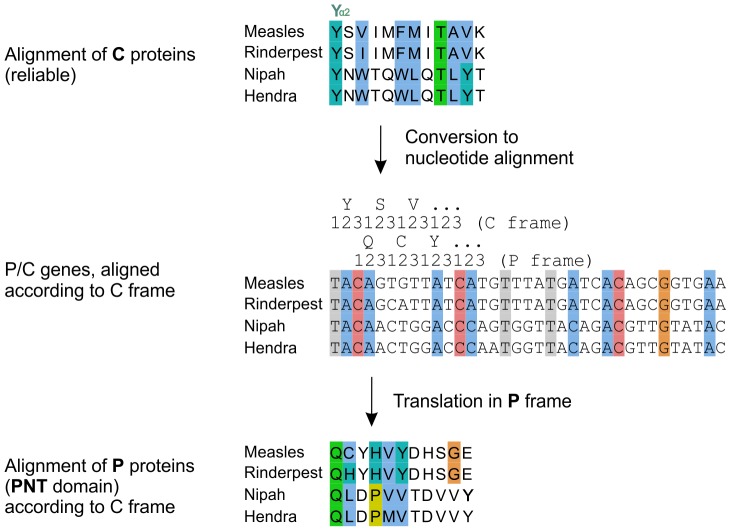
Procedure to reliably align the central region of PNT of the measles and Nipah groups by using a C frame alignment. Conventions are the same as in Figure 2. An alignment of C (top panel) is converted in a nucleotide alignment (middle panel) by using TranslatorX (see text), then translated into the P frame (bottom panel), yielding a reliable alignment of the PNT domain of P, which overlaps C. The nucleotide alignment of the P/C genes corresponding to the middle panel is in File S2.

**Figure 9:**
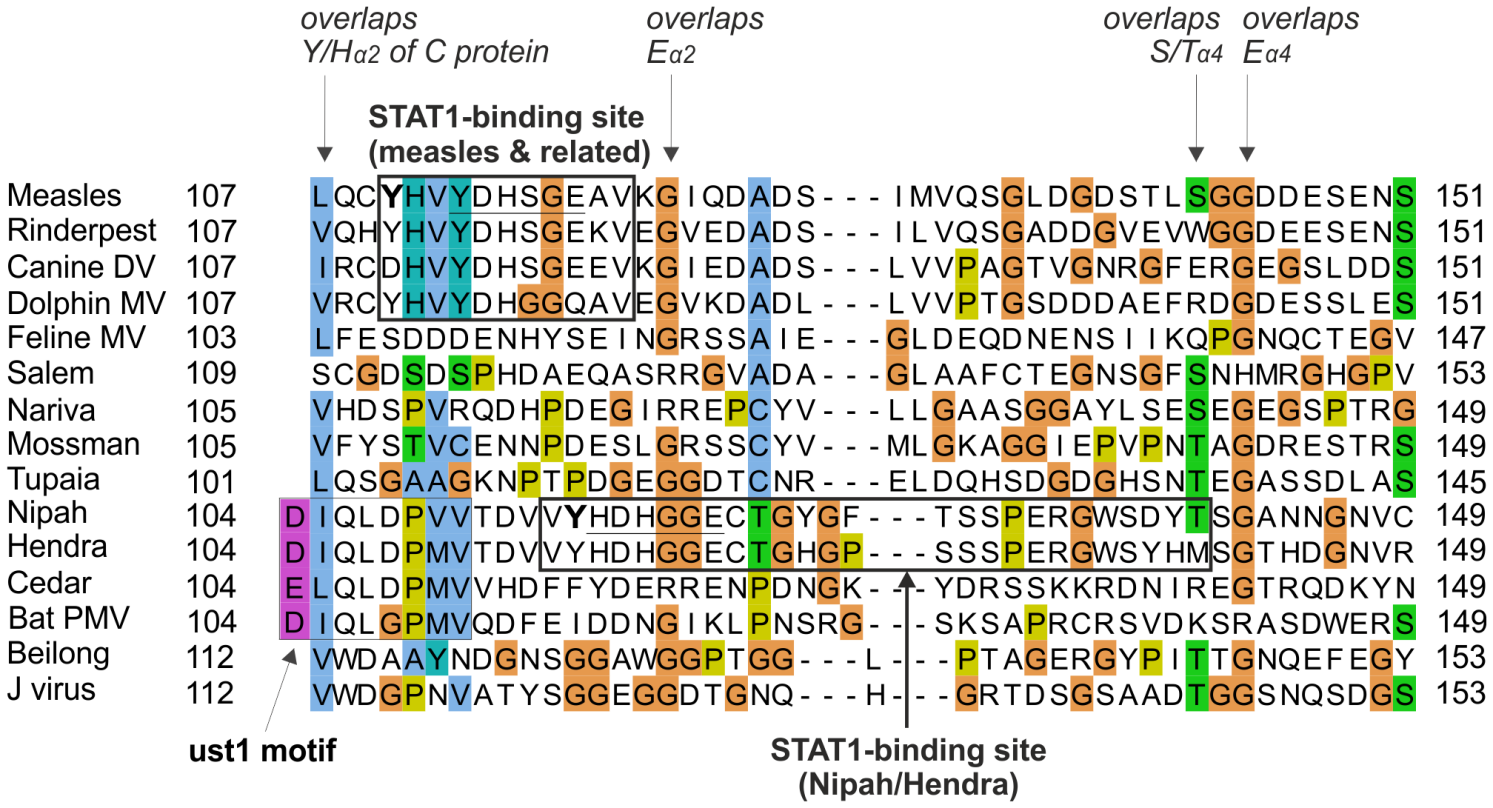
Alignment of the region of PNT of the measles and Nipah groups containing STAT1-binding sites, corrected by using the C frame. Conventions are the same as in Figure 2. This reliable alignment of PNT is based on an alignment of the C frame by the procedure described in Figure 8. The [Y/H]DH[S/G]GE motifs common to the STAT1-binding sites of *measles virus* and *Nipah virus* PNT are underlined. Note that contrary to the alignment of Figure 7, here they are not aligned together. Y110 of *measles virus* PNT and Y116 of *Nipah virus* PNT, which were suggested to be analogous (see text) are in bold.

We present in Figure 6 a summary of the structural and functional organization of PNT and C in the different taxa of *Paramyxovirinae*, to scale, with their functional motifs vis-à-vis of each other. PNT contains sequences that bind the protein STAT1 in several *morbilliviruses* (*measles virus* [55], [56], *canine distemper virus* [57], *Rinderpest virus* [60]) and *henipaviruses* (*Nipah virus* [58] and *Hendra virus* [59]). The region of PNT that contains these sites is highly variable in sequence (Figure 7), and thus its alignment is not reliable. In contrast, the overlapping region of C is well conserved, and its alignment reliable (Figure 5). Therefore, we used the C frame to construct a reliable alignment of PNT. We proceeded in two steps (see Methods). First, we used the amino acid alignment of the C proteins (Figure 8, top panel) to generate an alignment of the nucleotide sequences of the P/C gene (Figure 8, middle panel and File S2), using TranslatorX [89]. Second, we translated this nucleotide alignment into an amino acid alignment in the P frame (Figure 8, bottom panel). The resulting alignment of PNT of the measles and Nipah groups is presented in Figure 9.

### The STAT1-binding Sites of PNT of Nipah Virus and Measles Virus Overlap Different Regions of C and thus Probably Evolved Independently

From the reliable alignment of PNT corrected by using the C frame (Figure 9), we made three observations:

- The STAT1-binding sites of *measles virus* and *Nipah virus* PNT are conserved in sequence only in very closely related species (thick boxes in Figure 9). For instance, in PNT of *Feline morbillivirus*, which is more distantly related to *measles virus* than other *morbilliviruses*, only 2 aa out of 11 (E110 and I116) correspond to conservative substitutions with respect to the STAT1-binding motif of *measles virus* (Figure 9). Such a high number of non-conservative substitutions within a short peptide suggests that it may not bind STAT1.
- The STAT1-binding sites of *measles virus* and *Nipah virus* PNT are not aligned together (Figure 9) (although they overlap slightly, by 4aa), which indicates that they are encoded in different locations of the P/C gene. It is thus highly likely that they have originated independently (see Discussion).
- The STAT1-binding sites of *measles virus* and *Nipah virus* PNT have some limited sequence similarity, as reported earlier [58]: they share a [Y/H]DH[S/G]GE motif, underlined in Figures 7 and 9. However, this similarity is unlikely to be due to homologous descent, since the motifs are not aligned together in the reliable alignment of PNT (Figure 9). Likewise, the tyrosine residues immediately upstream of this motif (Y110 in *measles virus* PNT, critical for STAT1 inhibition [33], [55], [112], [113], and Y116 in *Nipah virus* PNT), which were perceived to occur in a similar sequence context [58], are not aligned together either in the reliable alignment of PNT (Figure 9), indicating that they are not homologous either.

Finally, we also noticed an 8aa motif (aa 104–111 in *Nipah virus*) conserved in the PNT of all *henipaviruses* (Figure 9, thin box). We called this motif ust1 (for “upstream of STAT1”). Its function is unknown, though aa 81–113 of *Nipah virus* P, which include ust1, are required for the synthesis of viral RNA [58]. We cannot exclude, however, the possibility that the conservation of ust1 is due to constraints imposed by the overlapping C frame.

### Functional Organization of the C Proteins in Relation to Their Sequence

We systematically examined mutational studies of *Paramyxovirinae* C and their phenotypic impact. The most relevant studies are in Table 2 and a more extensive list of studies is in Table S1. We found that very few conserved positions identified herein have been subjected to targeted mutagenesis; notable substitutions are indicated in bold in Figures 2 and 4.

**Table 2:** Selected experimental substitutions in C and their effect.

Group	Virus name	C ORF mutation	Functional effect(s) of mutation	References
MEASLES GROUP	Measles virus	R44G	Ablates nuclear localization	[29], [156]
		**S134Y**	Associated with temperature-sensitive vaccine virus	[114]
		Δ127–138 (deletion)	Ablated interaction with SHCBP1, reduced ability toinhibit minigenome replication	[115]
NIPAH GROUP	Nipah virus	No fine mutational data	–	
SENDAI GROUP	Sendai virus	C Δ10–15 (deletion)	Inability to interact with and modulate levels ofphosphorylated STAT1	[48]
		Series of deletions inaa149–157	Loss of nuclear translocation of Y1 by Ran-GTPase pathway	[120]
		K151A/**E153L**/R157L (Cm*)	Increased IFN-β induction and dsRNA production, induction ofantiviral state, increased CPE, apathogenic *in vivo*	[39], [127]
		K77A/D80A (Cm2), M139A/D142A (Cm4), **R173A**/E175A/E176A (Cm8)	Inability to bind STAT1, ablated ability to inhibit RNAsynthesis, decreased binding to viral polymerase (L protein)	[46], [118]
		K77R/D80A (Cm2’), D80A	Increased cytopathic effect, increased nuclear translocationof IRF3, increased IFN-β induction and production of dsRNA	[39]
		K151A/**E153A**/R154A (Cm5)	Attenuated virulence *in vivo*, inability to block IFN signaling,inability to inhibit replication, inability to skew STAT1/2phosphorylation and to bind STAT1, decreased bindingto L protein	[39], [46], [118], [127]
	Human parainfluenzavirus 1 (hPIV1)	R84G	Increased IFN-β production, increased IRF3 nucleartranslocation, reduced plaque sizes, non-temperaturesensitive mutation contributing to attenuation *in vivo*	[43], [157]
	Human parainfluenzavirus 3 (hPIV3)	CNΔ25, CNΔ50 (deletions)	Increased inhibition of viral RNA synthesis, suppression of viralreplication, decreased ability to block Type 1 IFN signaling	[121], [122]
		K3A, K6A, K12A,E16A, R24A	Increased inhibition of viral RNA synthesis, decreasedability to block Type 1 IFN signaling	[121], [122]
		aa 90–195 of C	Region required for STAT1 binding	[119]

These studies used either recombinant viruses, minigenome systems, or eukaryotic expression systems. Substituted residues that are conserved in a group are in bold. For a more comprehensive list of studies on *Paramyxovirinae* C, please see Table S1.

In the measles group, experimental substitutions have been performed mostly in the C-terminus of C. In a comparison of a temperature-sensitive strain of measles vaccine, AIK-C, with its parental strain, Edmonston [114], one of several substitutions identified, S134Y, occurs in the S/T_α4_ position conserved in the measles and Nipah groups (Figures 2 and 5) (Table 2). Although this particular substitution is not responsible for the temperature sensitive phenotype [114], we note that it is located within a 12aa peptide (aa 127–138) recently shown to inhibit the viral polymerase by interacting with SHCBP1 (Shc Src homology 2 domain-binding protein 1) [115]. This peptide, underlined in Figures 2 and 5, contains two other positions conserved in the measles/Nipah groups (a hydrophobic residue and E_α4_). Such conservation suggests that other viruses in the measles/Nipah groups may also bind SHCBP1 to block the viral polymerase. Finally, the role of the disordered N-terminus of *measles virus* C is poorly known, although it contributes to nuclear localization, which correlates with its ability to block IFN induction [29] (Table 2).

In the Nipah group, there are no fine mutational data published, but it is known that both the N-terminus and the C-terminus of *Nipah virus* C are required to inhibit minigenome replication [116].

In the Sendai group, experimental substitutions have delineated multiple residues in the C-terminus of C responsible for antagonizing both IFN induction and IFN signaling, and for regulating viral transcription and replication [46], [49], [117], [118] (Table 2 and Table S1). For both *Sendai virus* and hPIV3, the minimal region required for STAT1-binding corresponds to the structured, well-conserved C-terminus of C [117], [119]. Within that domain, aas 149–157 (corresponding roughly to basic region 2, underlined in Figure 4) are critical for nuclear translocation of the Y1 isoform of *Sendai virus* C, and may also play a role in the inhibition of type-I IFN-stimulated gene expression [120]. This region contains several conserved residues, suggesting that its function may be conserved in the Sendai group. Studies of the N-terminus of C in the Sendai group indicate that it also contributes to antagonizing the innate immune response and to regulate viral transcription and replication [121], [122] (Table 2 and Table S1). Taken together, these studies suggest that both the N- and C-terminus of Sendai group C proteins may need to act in coordinated fashion in order to perform their complete suite of antagonistic and regulatory functions.

### Experimental Characterization of one C Protein of the Measles/Nipah Group and of One C Protein of the Sendai Group

In order to check our predictions of structural organization, we attempted to characterize biophysically at least one C protein of the measles/Nipah groups and one of the Sendai group. We systematically tested, in the bacteria *E. coli,* the expression and solubility of the C proteins of all species in the measles, Nipah and Sendai groups (see Methods). We found that the C proteins of *tupaia paramyxovirus* (Tupaia PMV) and of hPIV1 were by far the best candidates, for the measles/Nipah groups and Sendai group respectively, in terms of yield and solubility (not shown). We expressed both proteins as hexahistidine-tagged N-terminal fusion proteins in *Escherichia coli* and purified them from the soluble fraction by immobilized metal affinity chromatography (IMAC) and size exclusion chromatography (SEC) (see Methods). Mass spectrometry confirmed that the C proteins had the exact expected mass. In SDS-PAGE analysis (Figure S1), hPIV1 C migrated at a notably larger size (∼31kD) than expected (25.9kD), while Tupaia PMV C migrated at ∼21kD, only slightly above the expected size (19.7kD). This anomalous migration may be caused by regions that are disordered or have a biased aa composition [123]. Accordingly, the N-terminus of both proteins is predicted disordered, and has a biased composition in the case of hPIV1 C.

**Figure 10:**
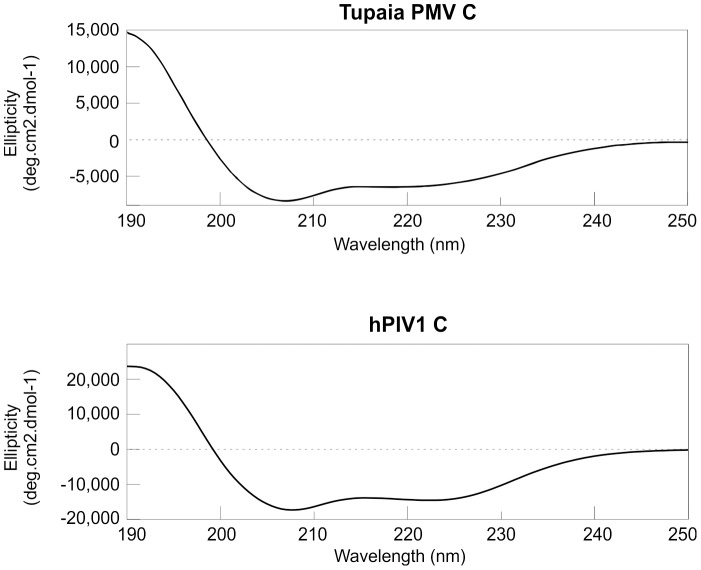
Circular Dichroism (CD) spectra of the C proteins of hPIV1 and Tupaia PMV.

**Figure 11:**
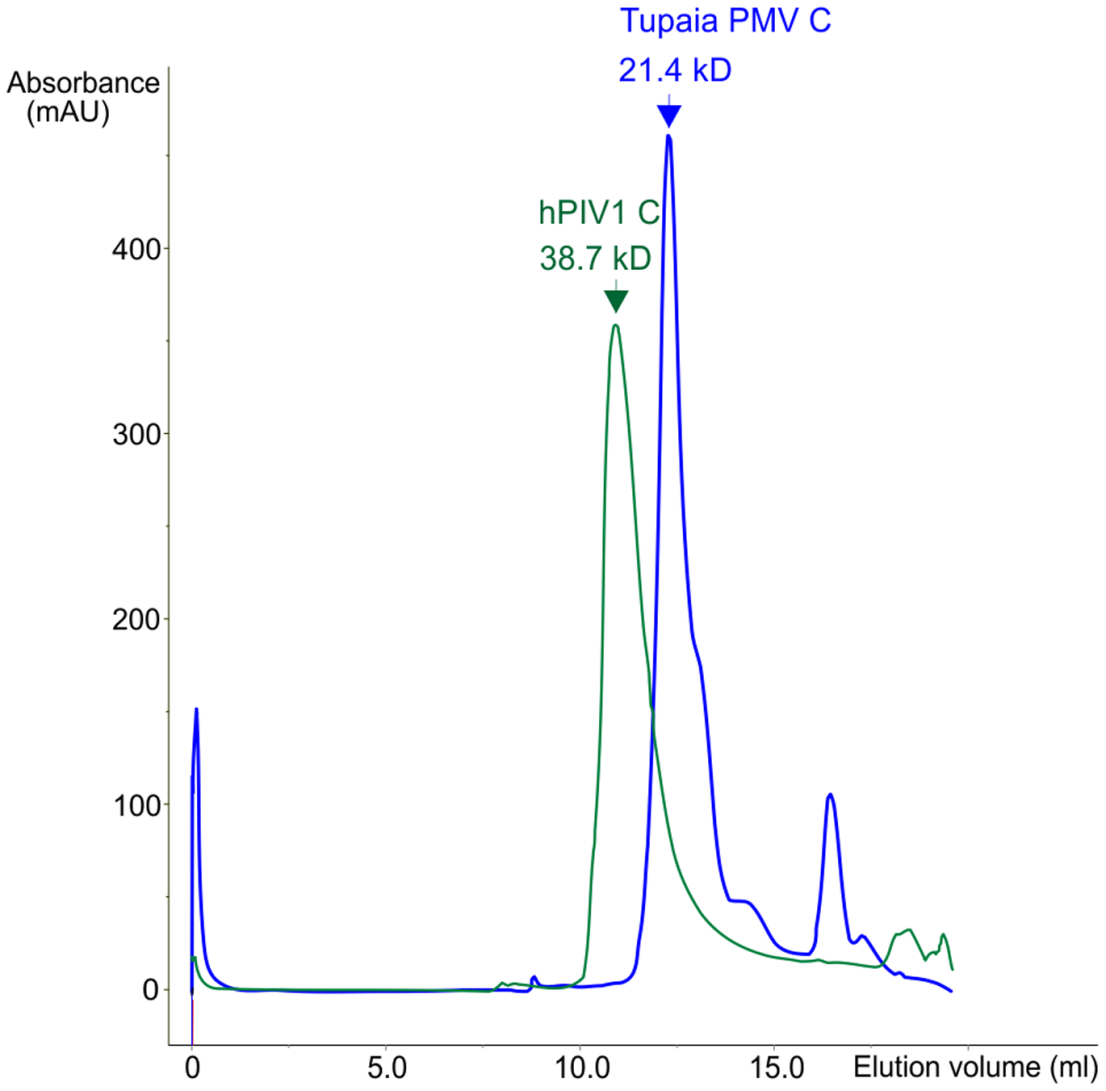
Size Exclusion Chromatography (SEC) on the C proteins of hPIV1 and Tupaia PMV. The curves represent two SEC purifications runs, on the same column and in similar conditions (see text): one for Tupaia PMV C (blue), and one for hPIV1 C (green).

We analyzed the secondary structure of the C proteins by Circular Dichroism (CD). The CD spectrum of both proteins (Figure 10) is typical of α-helical content [124], with two dips in ellipticity at around 208 and 222 nm. The estimated α-helical content was 57% for hPIV1 C and 33% for Tupaia PMV C (see Methods). We also examined the C proteins by analytical SEC (Figure 11). Tupaia PMV C elutes at an apparent molecular mass of 21.4 kDa, close to its theoretical mass of 19.7 KDa. In contrast, hPIV1 C elutes at a much larger MW (38.7 kDa) than expected (25.9 kDa). This discrepancy could correspond to an extended shape, or to self-association in a fast equilibrium between a monomeric and dimeric form (see below).

### Limited Proteolysis of hPIV1 C and Tupaia PMV C Confirms That they Have a Flexible N-terminus and a Structured C-terminus

**Figure 12:**
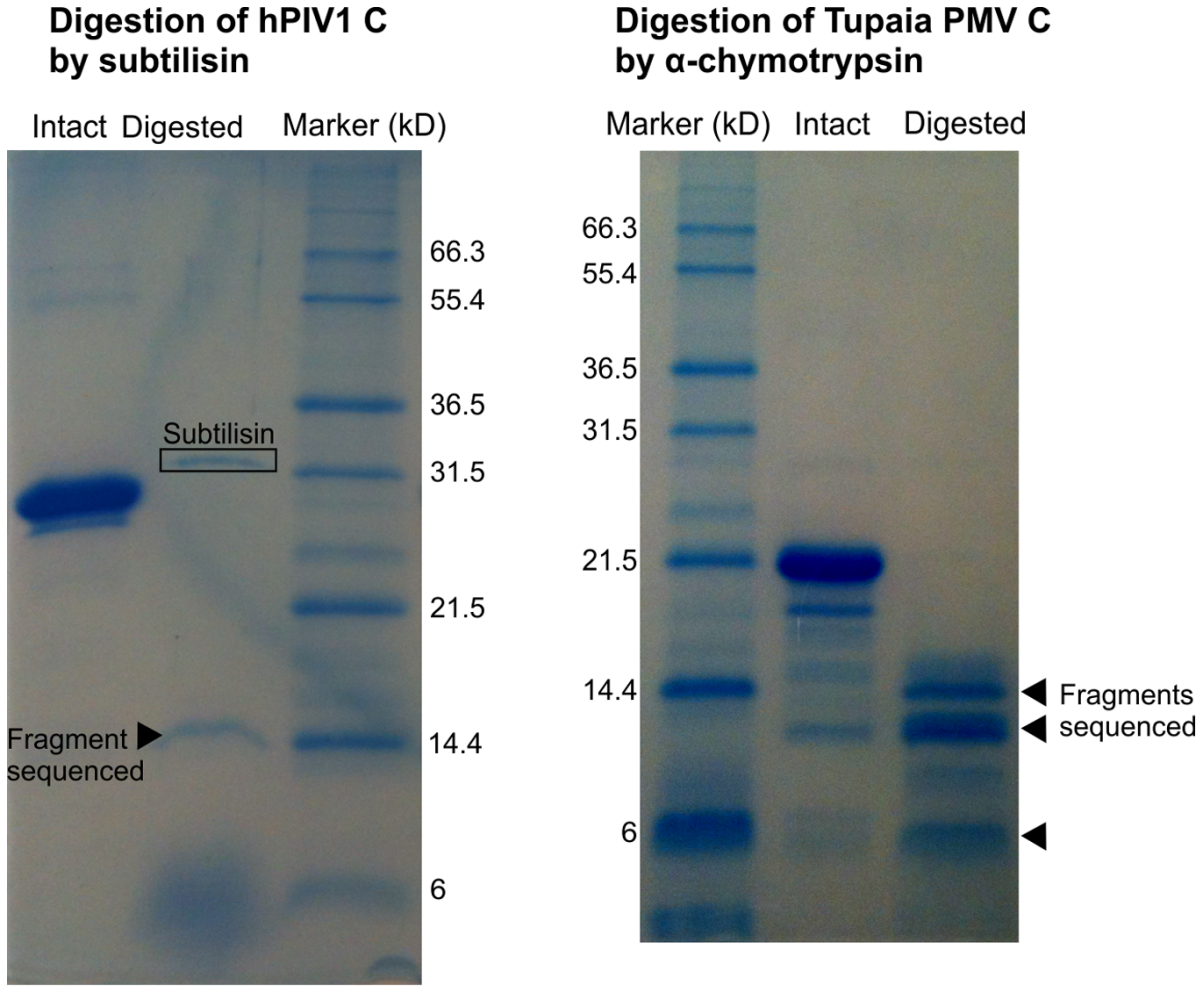
Limited proteolysis of the C proteins of hPIV1 and Tupaia PMV. Digestion profiles of hPIV1 C (left) and Tupaia PMV C (right), visualized by SDS-PAGE and Coomassie blue staining. Several fragments (arrowheads) were N-terminally sequenced. Their N-terminal sequences are underlined in Figures 2 and 4. α–chymotrypsin is not visible on the digestion profile of Tupaia PMV C.

We used limited proteolysis combined with N-terminal sequencing to probe the structural organization of the C proteins of hPIV1 and Tupaia PMV. We tested a range of proteases with different substrate requirements (see Methods), and identified fragments resistant to proteolysis, indicative of folded domains. Digestion of hPIV1 C by subtilisin yielded a stable degradation product of around 14 kD (Figure 12, left panel), whose N-terminal sequence, starting at aa 104, is underlined in Figure 4. The size of this fragment indicates that it comprises the whole C-terminus of C (expected size 14.16 kD), which corresponds well to our sequence predictions (Figure 4). These results are also coherent with cellular experiments that identified a proteolysis-sensitive N-terminus in the C’ proteins of *Sendai virus* [125]. We note that the presence of a long, disordered region in hPIV1 C is compatible with its high apparent molecular weight observed in SEC (see above) [126].

Digestion of the C protein of Tupaia PMV by α-chymotrypsin yielded a series of bands ranging from 14 kD to 6 kD (Figure 12, right panel); further digestion (not shown) yielded a single 6kD fragment. We obtained N-terminal sequences of the three most abundant fragments, of ∼14.4, 13, and 6 kD (arrows to the right of Figure 12). They start respectively at aa 30, 43 and 84. This pattern of proteolytic digestion indicates that Tupaia PMV C is composed of a disordered N-terminus and of an ordered C-terminus. This is compatible with our predictions, in which aa 1–56 are devoid of secondary structure (Figure 2) and aa 1–42 disordered, and in which a predicted loop, α_1–2_ (aa 81–92), could be accessible to proteolysis. The observed fragments of 14.4 and 13kD correspond exactly to C proteins where aa 1–29 and 1–43, respectively, have been digested, whereas the size of the smaller fragment (6kD) corresponds to aa 81–135, indicating that the last 18 C-terminal aa are digested upon extended proteolysis.

In summary, our experiments confirm that *in vitro*, the C proteins of hPIV1 and Tupaia PMV are predominantly α-helical and contain a disordered N-terminus, whose boundaries are in good agreement with our sequence-based predictions.

## Discussion

Substituting the conserved, charged residues we have identified herein should be a powerful way to dissect the function of C. Indeed, charged residues are often on the surface of proteins and thus their conservation is generally the result of functional constraints, rather than constraints imposed by a mere structural role. The power of this approach has been shown by studies on several regions of *respirovirus* C [39], [46], [127], and our thorough sequence analysis of the full-length C proteins of all *Paramyxovirinae* should greatly extend its applicability. In addition, knowing the structural organization of C will allow the design of deletions that have less risk of disrupting its three-dimensional structure.

### A Common Origin of the C Proteins?

The C proteins of the Sendai group have no detectable sequence similarity with those of the measles/Nipah groups. However, we consider it unlikely that they have an independent origin, because they are located in the same region of the P gene, in the same frame relative to P, and have a similar structural organization and several similar functions [118], [128], [129]. Thus we consider that all C proteins most probably have a common origin, as proposed earlier [67], [130]. The absence of a C protein in the mumps group is probably due to a loss in the ancestor of that group, since the Sendai group, which has a C protein, is basal in a phylogeny of the P gene [105]. This common origin would imply that in *Sendai virus*, it is the Y1 isoform of C that is the equivalent of C of the measles/Nipah groups, because their start codon have the same location immediately upstream of the soyuz1 motif of the P frame (Figure 6; compare also Figure 4 and Figure 3). Therefore, the C and C’ proteins of *Sendai virus* would have presumably originated by mutations creating new, alternative start codons upstream of Y1. A common origin of *Paramyxovirinae* C proteins would also imply that the basic regions in the N-terminus of C have originated independently in the Sendai and Nipah groups, since they occupy different positions with respect to soyuz1 (Figure 6).

### Which Frame Originated Earlier, PNT or C?

Overlapping genes typically encode an ancestral frame and a novel frame originated by overprinting it (see Introduction). Our analyses in this work and in an earlier study [14] suggest that the C and PNT frames were probably both present in the ancestor of *Paramyxovirinae*, making it impossible to conclude which frame is ancestral on the basis of phylogeny. Analysis of codon usage [131] cannot determine which frame is ancestral either, because the codon usages of PNT and C are indistinguishable in *Paramyxovirinae* (Angelo Pavesi, personal communication). However, functional considerations suggest that the PNT frame originated earlier, since it is indispensable to viral replication *in vitro* [2], [132], unlike C [20], [133], [134]. The ancestry of PNT is supported by a comparison with families related to *Paramyxovirinae* (*Mononegavirales*). Most *Mononegavirales* also encode P proteins with a disordered N-terminus [14], [135]; at least in *Rhabdoviridae*, this N-terminus has the same function as *Paramyxovirinae* PNT, i.e. preventing the nucleoprotein from self-assembling illegitimately [136]–[139]. Thus, it is reasonable to speculate that the P of the ancestral *Mononegavirales* already had a disordered N-terminus, which was overprinted by C in the ancestor of *Paramyxovirinae*.

### Convergent Evolution between the STAT1-binding Sites of *measles virus* and *Nipah virus*?

The STAT1-binding sites of *measles virus* and *Nipah virus* do not align together in the reliable alignment of PNT, generated using the C frame (Figure 9). This strongly suggests that they have originated independently. Alternatively, since they overlap by 4aa (Figure 9), these STAT1-binding sites might, in theory, have originated from a common, short peptide, providing some STAT1-blocking capability, and later have extended respectively upstream and downstream of PNT. However, this scenario is not parsimonious because it would imply several losses in the lineages separating *measles virus* and *Nipah virus*. Also, the common 4aa stretch is chemically very different in both viruses (G_117_EAV in *measles virus* and V_115_YHD in *Nipah virus*, Figure 9). We thus consider it most likely that the STAT1-binding sites of *measles virus* and *Nipah virus* have originated independently.

Their limited sequence similarity (they share an [Y/H]DH[S/G]GE motif, underlined in Figure 9) would thus not be the result of homologous descent, but could instead result either from convergent evolution (owing to a common mechanism), or from random chance. Convergent evolution seems a definite possibility, since the mechanisms by which PNT acts are somewhat similar in both viruses (PNT interferes with the phosphorylation of cytoplasmic STAT1) [55], [58], [112], [140], and since the PNT of both viruses bind a similar part of STAT1 [141].

### The P/C Gene Exemplifies Three Keys to the Evolutionary Paradox of Overlapping Genes

Overlapping genes are an evolutionary paradox, because they simultaneously encode two proteins whose freedom to mutate is constrained by each other, which should severely reduce the ability of the virus to adapt [75]–[81].

**Figure 13:**
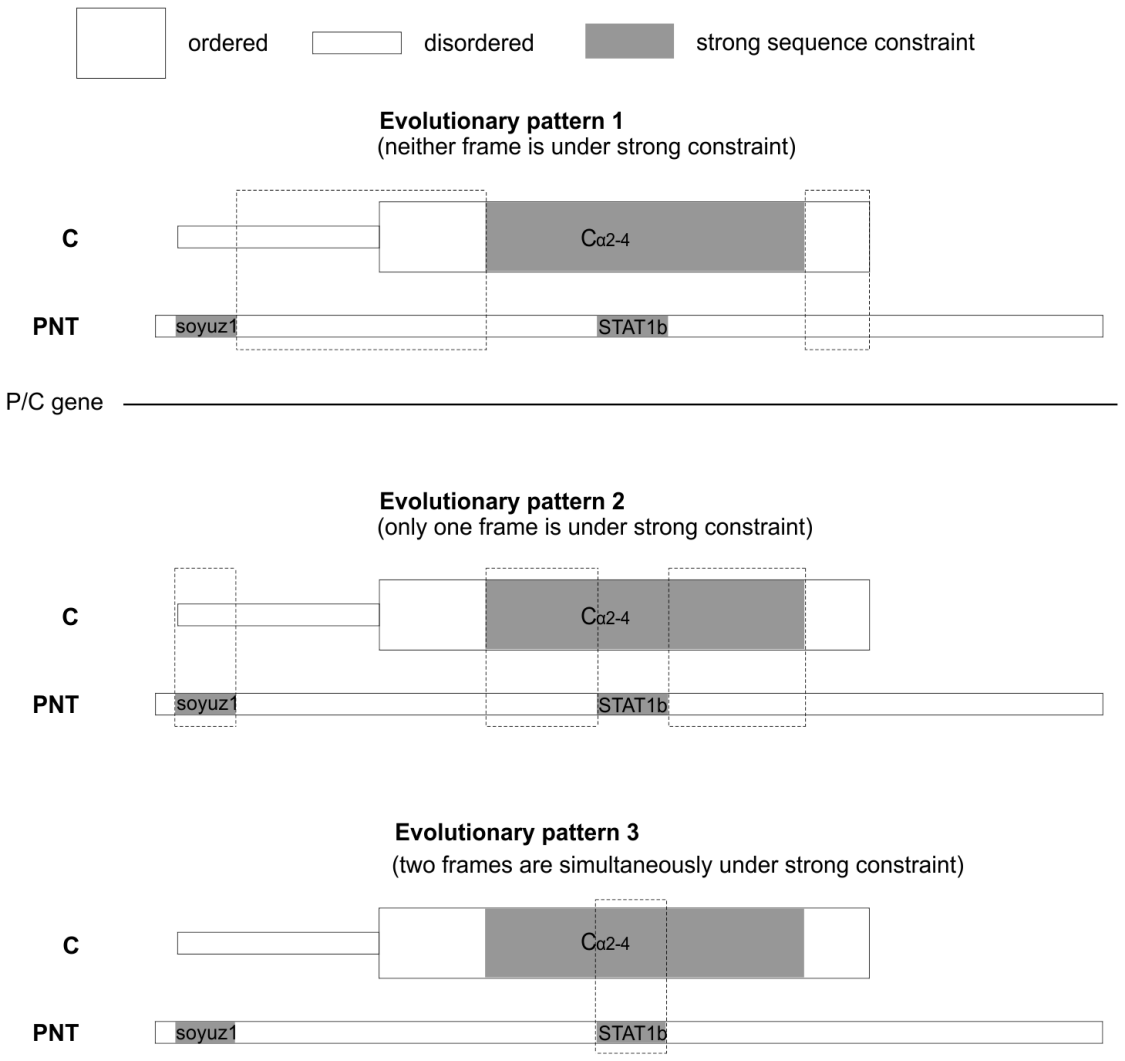
Three patterns of sequence constraints in the overlapping frames P and C. PNT and C are represented vis-à-vis of each other with same conventions as in Figure 6. Sequence constraints of PNT and C were estimated by their sequence variability.

A first key to the paradox has been suggested earlier [67], [77], [78], [142]–[146]: overlapping genes frequently encode an “ancillary” frame that can tolerate a higher substitution rate than the other, “dominant” frame; the ancillary frame is often structurally disordered [68]. Accordingly, a previous sequence analysis of *Sendai virus* indicated that PNT and C are generally not both under strong constraint [142]; rather, the N-terminus of PNT is markedly more conserved than that of C, whereas the C-terminus of PNT is markedly more conserved than that of C [142]. This is also the case for most of the PNT and C of measles and Nipah virus (Figure 13, evolutionary pattern 1 or 2), with the exception of the region corresponding to the STAT1-binding sites of PNT (see below).

A second key to the paradox of overlapping genes is that it may be beneficial for a virus, under certain conditions, to encode functional motifs simultaneously by using overlapping frames [147]. Initially, we were very surprised to discover that a region of the P/C gene encodes simultaneously, in different frames, two well-conserved regions: the STAT1-binding motif of PNT, and the α2–α4 region of C (Figure 13, evolutionary pattern 3). Intuitively, this arrangement seems to dramatically restrict the capacity of the virus to mutate and to escape host defenses. We were all the more surprised that this arrangement originated twice independently, in *measles virus* and in *Nipah virus* (see Figure 6). This seems beyond coincidence, and strongly suggests that the loss of fitness of the virus due to its reduced ability to mutate is compensated by an evolutionary advantage. In fact, this phenomenon had been predicted on the basis of mathematical modeling [147]. Given a high mutation rate, it may be advantageous to encode crucial functional motifs in overlapping frames (provided that they are short), because the superposition of critical amino acids reduce the number of vulnerable positions in the genome. The conditions of application of the model are met here: RNA viruses have one of the highest mutation rate of all organisms [148], and the STAT1-binding sites are short (10–26aa). It will be interesting to investigate whether this evolutionary pattern, in which two reading frames are both under strong constraint, is common in viruses, and whether it does entail a selective advantage. The genome of *Hepatitis B virus*, for instance, also contains short regions where both the overlapping Polymerase and Glycoprotein frames are under strong constraint [149], [150]. A recent innovative methodology that combines experimental and computational approaches [151] could help to tease out the different factors (structural, functional and co-evolutionary) constraining overlapping motifs.

Finally, a third key to the paradox of overlapping genes is that they provide a regulatory advantage that may offset the increased constraints they impose on the virus, by encoding two proteins that are co-regulated and have complementary functions [131]. For instance, the expression levels of the C and V proteins of Nipah or measles viruses are co-regulated, since they are transcribed from the same gene transcription unit; in addition, their roles are complementary, since together they inhibit both viral RNA synthesis and type I IFN induction, enabling an efficient block of the first stage of the host antiviral response [15], [17], [20], [24], [152]. In the same vein, the expression of C and P is also co-regulated and they have complementary effects on viral transcription, mediated by binding the same cellular protein, SHCBP1 [115].

### Conclusion

In conclusion, we predict that the C proteins of the Sendai group and of the measles/Nipah groups will have the same structural fold, testifying to a common origin, and that this fold will be a previously unobserved one, in keeping with their *de novo* origin [68].

## Supporting Information

Figure S1 **Purification of the C proteins of hPIV1 and Tupaia PMV.** The purifications are visualized by Coomassie blue-stained SDS-PAGE.
(TIF)

Table S1 **Effect of experimental substitutions in** ***Paramyxovirinae*** **C proteins.**
(DOCX)

File S1 **Multiple sequence alignment of the C proteins of the measles, Nipah, and Sendai groups.**
(DOC)

File S2 **Multiple sequence alignment of the P/C genes of the measles and Nipah groups, based on an alignment of the C proteins**
(DOC)
