# Supplementary figures and images for: Evolution and Structural Organization of the C Proteins of Paramyxovirinae
Source: PLoS One. 2014 Feb 25;9(2):e90003. doi: 10.1371/journal.pone.0090003 (PMC3934983; doi:10.1371/journal.pone.0090003)

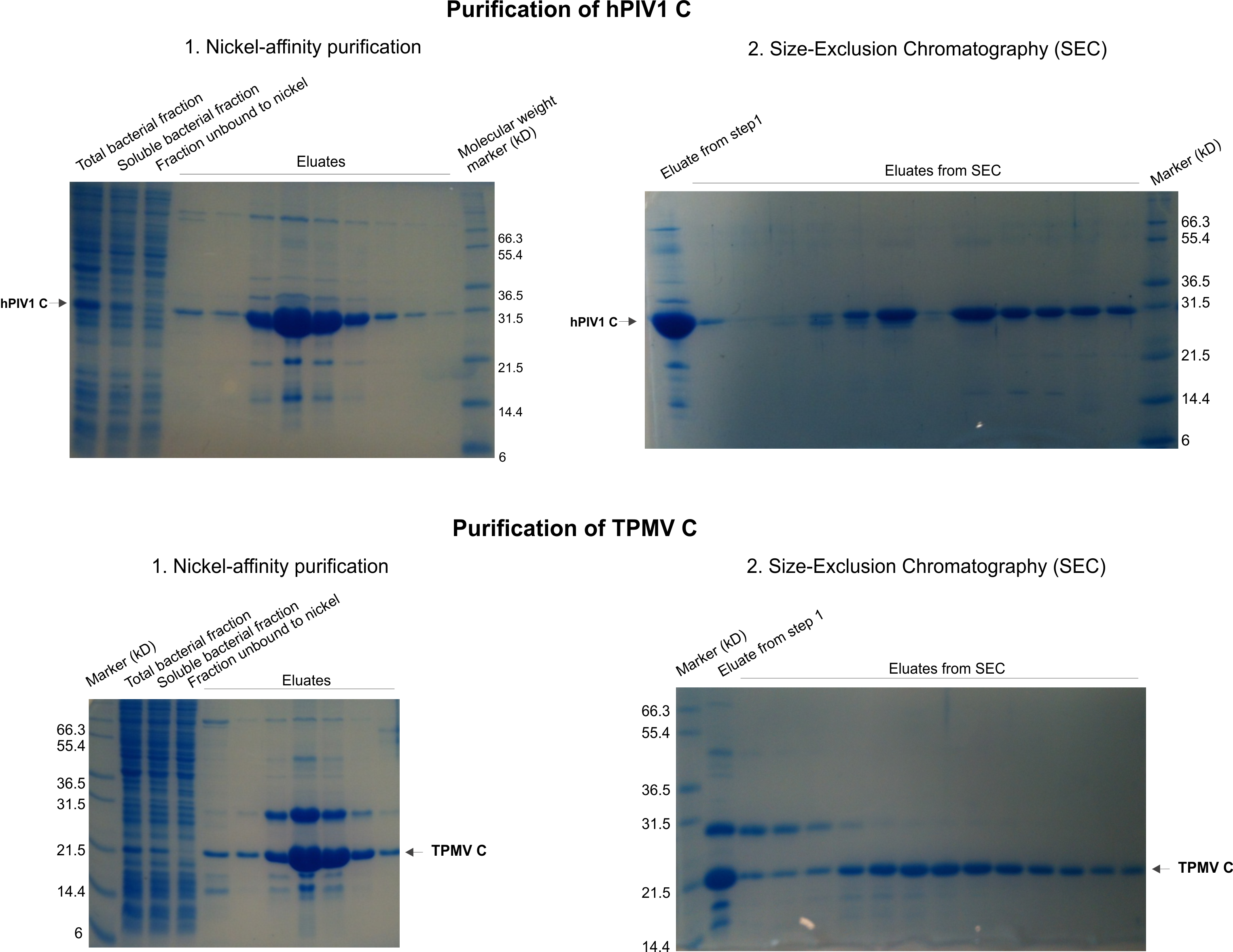

Supplement: Figure S1 — Purification of the C proteins of hPIV1 and Tupaia PMV. The purifications are visualized by Coomassie blue-stained SDS-PAGE. (TIF) [file pone.0090003.s001.tif]
