# Supplementary material for: Evolution and Structural Organization of the C Proteins of Paramyxovirinae
Source: PLoS One. 2014 Feb 25;9(2):e90003. doi: 10.1371/journal.pone.0090003 (PMC3934983; doi:10.1371/journal.pone.0090003)
Supplement: Table S1 — Effect of experimental substitutions in Paramyxovirinae C proteins. (DOCX) [file pone.0090003.s002.docx]

**Supporting Table 1. Effect of experimental substitutions in *Paramyxovirinae* C proteins**

The studies below used either recombinant viruses, minigenome systems, or eukaryotic expression systems. Substituted residues that are conserved in a group are in bold. Particularly relevant substitutions are in Table 2.

| **Group** | **Virus name** | **C ORF mutation** | **Functional effect(s) of mutation** | **References** |
| --- | --- | --- | --- | --- |
|  | Measles virus | Silencing or exclusion of C ORF | Attenuated virulence, lack of rash, reduced virus replication efficiency *in vivo*, increased IFN-β induction, induced cell growth arrest, activation of PKR & IRF1 through increased accumulation of double-stranded viral RNA | [[1-10](#_ENREF_1)] |
| MEASLES GROUP |  | R44G | Ablates nuclear localization | [[11](#_ENREF_11),[12](#_ENREF_12)] |
|  |  | R44G,I147T, S158🡪(STOP) (C157), S166P | Increased viral RNA transcription and replication | [[3](#_ENREF_3),[13](#_ENREF_13),[14](#_ENREF_14)] |
|  |  | R44G, S158🡪(STOP) (C157), | Reduces IFN-β inhibition, increases IRF3 activation | [[3](#_ENREF_3),[11](#_ENREF_11)] |
|  |  | V102A ,K111M | Associated with attenuated virus replication and virulence | [[15](#_ENREF_15),[16](#_ENREF_16)] |
|  |  | M104T | Associated with Moraten, Rubeovax, and Schwartz vaccine strains | [[17](#_ENREF_17)] |
|  |  | **S134Y** | Associated with temperature-sensitive vaccine virus | [[18](#_ENREF_18)] |
|  |  | Δ127-138 (deletion) | Ablated interaction with SHCBP1, reduced ability to inhibit minigenome replication, reduced viral titers | [[19](#_ENREF_19)] |
|  | Rinderpest virus | Silencing or exclusion of C ORF | Increased IFN-β induction, viral transcriptional defect | [[20](#_ENREF_20),[21](#_ENREF_21)] |
|  | Canine distemper virus | Silencing or exclusion of C ORF | Decreased rash *in vivo* | [[22](#_ENREF_22)] |
| NIPAH GROUP | Nipah virus | Silencing or exclusion of C ORF | Attenuated virulence and reduced virus replication efficiency *in vivo*, increased IFN-β and chemokine induction, increased early viral RNA transcription and replication | [[23-26](#_ENREF_23)] |
| SENDAI GROUP | Sendai virus | (C’/C) Silencing or exclusion of C ORF | Dysregulation of genome to antigenome ratio, inability to increase STAT-1 ubiqitination or decrease bulk STAT1 levels, induction of apoptosis, ablates inhibition of IFN-β induction via RIG-I, restricted *in vitro* and *in vivo* replication, decreased ability to block IFN signaling | [[27-33](#_ENREF_27)]{Irie, 2010 #415} |
|  |  | 4C(-) Silenced/excluded | Increase in double-stranded RNA (dsRNA) production and PKR activation, dysregulation of genome to antigenome ratio, increased nuclear translocation of IRF3, increased cytopathic effect (CPE), restricted *in vitro* and *in vivo* replication, decreased ability to block IFN signaling | [[27](#_ENREF_27),[30-32](#_ENREF_30),[34](#_ENREF_34)] |
|  |  | C^1-23^ region | Plasma membrane targeting signal | [[35](#_ENREF_35)] |
|  |  | C ∆10-15 (deletion) | Inability to interact with and modulate levels of phosphorylated STAT1 | [[36](#_ENREF_36)] |
|  |  | C^24-204^ region | Region required for IFN-β inhibition | [[29](#_ENREF_29)] |
|  |  | C 1-126 (deletion), C 1-67/181🡪STOP, C 1-67/190🡪 STOP | Inability to block establishment of antiviral state, ablated ability to inhibit viral transcription/replication | [[37](#_ENREF_37)] |
|  |  | K77A/D80A (Cm2), M139A/D142A (Cm4), **R173A**/E175A/E176A (Cm8) | Inability to bind STAT1, ablated ability to inhibit RNA synthesis, decreased binding to viral polymerase (L protein ) | [[38](#_ENREF_38),[39](#_ENREF_39)] |
|  |  | E129A/E132A (C4), R157A (C6) | Decreased binding to L protein | [[39](#_ENREF_39)] |
|  |  | E114A/E115A (Cm3), | Loss of binding to STAT1 | [[38](#_ENREF_38)] |
|  |  | M139A/D142A (Cm4), L156A (Cm6), | Loss of binding to STAT1, decreased ability to inhibit RNA synthesis | [[38](#_ENREF_38)] |
|  |  | K77R/D80A (Cm2’), D80A | Increased CPE, increased nuclear translocation of IRF3, Increased IFN-β induction, production of dsRNA | [[30](#_ENREF_30)] |
|  |  | M139T/D142A (Cm4’) | Increased CPE | [[30](#_ENREF_30)] |
|  |  | Series of deletions in aa 149-157 | Loss of for nuclear translocation of Y1 by Ran-GTPase pathway | [[40](#_ENREF_40)] |
|  |  | K151A/**E153A**/R154A (Cm5) | Attenuated virulence *in vivo*, inability to block IFN signaling, inability to inhibit replication, inability to skew STAT1/2 phosphorylation and to bind STAT1, decreased binding to L protein | [[30](#_ENREF_30),[38](#_ENREF_38),[39](#_ENREF_39),[41](#_ENREF_41)] |
|  |  | K151A/**E153L**/R157L (Cm*) | Increased IFN-β induction, dsRNA production, induction of antiviral state, increased CPE, apathogenic *in vivo* | [[30](#_ENREF_30),[41](#_ENREF_41)] |
|  |  | A194🡪STOP(d194), V189🡪STOP (d189), E184🡪STOP(d184), L181🡪STOP (d181), & A126🡪STOP (d126) COOH-terminal truncations | Inability to interact with Alix, decreased virus budding | [[42](#_ENREF_42)] |
|  | Human parainfluenza virus 1 (hPIV1) | Silenced/excluded, | Increased formation of dsRNA, increased IRF3, NFκB, and PKR activation, increased IFN-β production and signaling, increased viral RNA synthesis, inhibited viral protein synthesis, attenuation *in vivo*, induction of apoptosis | [[43-45](#_ENREF_43)] |
|  |  | F170S | Increased formation of dsRNA, increased IRF3, NFκB, and PKR activation, increased IFN-β production and signaling, increased viral RNA synthesis, inhibited viral protein synthesis, attenuated *in vivo*, weakened STAT1 interaction | [[44-48](#_ENREF_44)] |
|  |  | ∆170 (RDF🡪S) | Attenuated *in vivo*, increased IFN- production and signaling, increased IRF3 activation and nuclear translocation | [[44](#_ENREF_44),[46](#_ENREF_46),[49](#_ENREF_49)] |
|  |  | R84G | Increased IFN-β production, increased IRF3 nuclear translocation, reduced plaque sizes, non-temperature sensitive mutation contributing to attenuation *in vivo* | [[44](#_ENREF_44),[50](#_ENREF_50)] |
|  | Human parainfluenza virus 3 (hPIV3) | Silenced/excluded | Attenuated *in vivo* and *in vitro*, increased viral RNA synthesis | [[51](#_ENREF_51),[52](#_ENREF_52)] |
|  |  | rF164S | Attenuated *in vivo* (upper respiratory tract) | [[51](#_ENREF_51)] |
|  |  | CN∆25, CN∆50 (deletions) | Increased inhibition of viral RNA synthesis, suppression of hPIV3 and RSV viral replication, decreased ability to block type 1 IFN signaling | [[53](#_ENREF_53),[54](#_ENREF_54)] |
|  |  | K3A, K6A, K12A, E16A, R24A | Increased inhibition of viral RNA synthesis, decreased ability to block Type 1 IFN signaling | [[53](#_ENREF_53),[54](#_ENREF_54)] |
|  |  | S7A (Cm1) | Increase in C protein phosphorylation levels, increased IRF3 activation, IFN-β transcription | [[55](#_ENREF_55),[56](#_ENREF_56)] |
|  |  | S47AT48A (Cm3) | Increase in C protein phosphorylation levels, increased IRF3 activation, IFN-β transcription, increased ability to resist antiviral state | [[55](#_ENREF_55),[56](#_ENREF_56)] |
|  |  | S81A (Cm4) | Increase in C protein phosphorylation levels, variable inhibition of viral RNA synthesis, increased sensitivity to antiviral state, decreased IRF3 nuclear translocation | [[55](#_ENREF_55),[56](#_ENREF_56)] |
|  |  | S22A | Increase in C protein phosphorylation levels | [[56](#_ENREF_56)] |
|  |  | C∆65-75 (deletion) | Decreased inhibition of viral RNA transcription | [[52](#_ENREF_52)] |
|  |  | C^90-195^ region | Region required for STAT1 binding | [[57](#_ENREF_57)] |
|  |  | C^97-195^ region | Region required for GRB-2 binding | [[57](#_ENREF_57)] |

1. Takeuchi K, Takeda M, Miyajima N, Ami Y, Nagata N, et al. (2005) Stringent requirement for the C protein of wild-type measles virus for growth both in vitro and in macaques. J Virol 79: 7838-7844.

2. Escoffier C, Manie S, Vincent S, Muller CP, Billeter M, et al. (1999) Nonstructural C protein is required for efficient measles virus replication in human peripheral blood cells. J Virol 73: 1695-1698.

3. Nakatsu Y, Takeda M, Ohno S, Shirogane Y, Iwasaki M, et al. (2008) Measles virus circumvents the host interferon response by different actions of the C and V proteins. J Virol 82: 8296-8306.

4. Nakatsu Y, Takeda M, Ohno S, Koga R, Yanagi Y (2006) Translational inhibition and increased interferon induction in cells infected with C protein-deficient measles virus. J Virol 80: 11861-11867.

5. Yokota S, Okabayashi T, Fujii N (2011) Measles virus C protein suppresses gamma-activated factor formation and virus-induced cell growth arrest. Virology 414: 74-82.

6. Valsamakis A, Schneider H, Auwaerter PG, Kaneshima H, Billeter MA, et al. (1998) Recombinant measles viruses with mutations in the C, V, or F gene have altered growth phenotypes in vivo. J Virol 72: 7754-7761.

7. Patterson JB, Thomas D, Lewicki H, Billeter MA, Oldstone MBA (2000) V and C proteins of measles virus function as virulence factors in vivo. Virology 267: 80-89.

8. Toth AM, Devaux P, Cattaneo R, Samuel CE (2009) Protein kinase PKR mediates the apoptosis induction and growth restriction phenotypes of C protein-deficient measles virus. J Virol 83: 961-968.

9. Devaux P, Hodge G, McChesney MB, Cattaneo R (2008) Attenuation of V- or C-defective measles viruses: infection control by the inflammatory and interferon responses of rhesus monkeys. J Virol 82: 5359-5367.

10. Pfaller CK, Radeke MJ, Cattaneo R, Samuel CE (2013) Measles Virus C Protein Impairs Production of Defective Copyback Double-stranded Viral RNA and Activation of Protein Kinase R. J Virol.

11. Sparrer KM, Pfaller CK, Conzelmann KK (2012) Measles virus C protein interferes with Beta interferon transcription in the nucleus. J Virol 86: 796-805.

12. Nishie T, Nagata K, Takeuchi K (2007) The C protein of wild-type measles virus has the ability to shuttle between the nucleus and the cytoplasm. Microbes Infect 9: 344-354.

13. Bankamp B, Wilson J, Bellini WJ, Rota PA (2005) Identification of naturally occurring amino acid variations that affect the ability of the measles virus C protein to regulate genome replication and transcription. Virology 336: 120-129.

14. Reutter GL, Cortese-Grogan C, Wilson J, Moyer SA (2001) Mutations in the measles virus C protein that up regulate viral RNA synthesis. Virology 285: 100-109.

15. Takeda M, Kato A, Kobune F, Sakata H, Li Y, et al. (1998) Measles virus attenuation associated with transcriptional impediment and a few amino acid changes in the polymerase and accessory proteins. J Virol 72: 8690-8696.

16. Bankamp B, Hodge G, McChesney MB, Bellini WJ, Rota PA (2008) Genetic changes that affect the virulence of measles virus in a rhesus macaque model. Virology 373: 39-50.

17. Parks CL, Lerch RA, Walpita P, Wang HP, Sidhu MS, et al. (2001) Comparison of predicted amino acid sequences of measles virus strains in the Edmonston vaccine lineage. J Virol 75: 910-920.

18. Komase K, Nakayama T, Iijima M, Miki K, Kawanishi R, et al. (2006) The phosphoprotein of attenuated measles AIK-C vaccine strain contributes to its temperature-sensitive phenotype. Vaccine 24: 826-834.

19. Ito M, Iwasaki M, Takeda M, Nakamura T, Yanagi Y, et al. (2013) Measles virus non-structural C protein modulates viral RNA polymerase activity by interacting with a host protein SHCBP1. J Virol.

20. Boxer EL, Nanda SK, Baron MD (2009) The rinderpest virus non-structural C protein blocks the induction of type 1 interferon. Virology 385: 134-142.

21. Baron MD, Barrett T (2000) Rinderpest viruses lacking the C and V proteins show specific defects in growth and transcription of viral RNAs. J Virol 74: 2603-2611.

22. von Messling V, Svitek N, Cattaneo R (2006) Receptor (SLAM [CD150]) recognition and the V protein sustain swift lymphocyte-based invasion of mucosal tissue and lymphatic organs by a morbillivirus. J Virol 80: 6084-6092.

23. Mathieu C, Guillaume V, Volchkova VA, Pohl C, Jacquot F, et al. (2012) Nonstructural Nipah virus C protein regulates both the early host proinflammatory response and viral virulence. J Virol.

24. Ciancanelli MJ, Volchkova VA, Shaw ML, Volchkov VE, Basler CF (2009) Nipah virus sequesters inactive STAT1 in the nucleus via a P gene-encoded mechanism. J Virol 83: 7828-7841.

25. Lo MK, Peeples ME, Bellini WJ, Nichol ST, Rota PA, et al. (2012) Distinct and overlapping roles of nipah virus p gene products in modulating the human endothelial cell antiviral response. PLOS ONE 7: e47790.

26. Sleeman K, Bankamp B, Hummel KB, Lo MK, Bellini WJ, et al. (2008) The C, V and W proteins of Nipah virus inhibit minigenome replication. J Gen Virol 89: 1300-1308.

27. Irie T, Nagata N, Yoshida T, Sakaguchi T (2008) Paramyxovirus Sendai virus C proteins are essential for maintenance of negative-sense RNA genome in virus particles. Virology 374: 495-505.

28. Garcin D, Marq JB, Strahle L, le Mercier P, Kolakofsky D (2002) All four Sendai Virus C proteins bind Stat1, but only the larger forms also induce its mono-ubiquitination and degradation. Virology 295: 256-265.

29. Strahle L, Marq JB, Brini A, Hausmann S, Kolakofsky D, et al. (2007) Activation of the beta interferon promoter by unnatural Sendai virus infection requires RIG-I and is inhibited by viral C proteins. J Virol 81: 12227-12237.

30. Irie T, Nagata N, Igarashi T, Okamoto I, Sakaguchi T (2010) Conserved charged amino acids within Sendai virus C protein play multiple roles in the evasion of innate immune responses. PLoS One 5: e10719.

31. Kurotani A, Kiyotani K, Kato A, Shioda T, Sakai Y, et al. (1998) Sendai virus C proteins are categorically nonessential gene products but silencing their expression severely impairs viral replication and pathogenesis. Genes Cells 3: 111-124.

32. Gotoh B, Takeuchi K, Komatsu T, Yokoo J, Kimura Y, et al. (1999) Knockout of the Sendai virus C gene eliminates the viral ability to prevent the interferon-alpha/beta-mediated responses. FEBS Lett 459: 205-210.

33. Irie T, Okamoto I, Yoshida A, Nagai Y, Sakaguchi T (2013) Sendai virus C proteins regulate viral genome and antigenome synthesis to dictate the negative genome polarity. J Virol.

34. Takeuchi K, Komatsu T, Kitagawa Y, Sada K, Gotoh B (2008) Sendai virus C protein plays a role in restricting PKR activation by limiting the generation of intracellular double-stranded RNA. J Virol 82: 10102-10110.

35. Marq JB, Brini A, Kolakofsky D, Garcin D (2007) Targeting of the Sendai virus C protein to the plasma membrane via a peptide-only membrane anchor. J Virol 81: 3187-3197.

36. Garcin D, Marq JB, Goodbourn S, Kolakofsky D (2003) The amino-terminal extensions of the longer Sendai virus C proteins modulate pY701-Stat1 and bulk Stat1 levels independently of interferon signaling. J Virol 77: 2321-2329.

37. Kato A, Ohnishi Y, Hishiyama M, Kohase M, Saito S, et al. (2002) The amino-terminal half of Sendai virus C protein is not responsible for either counteracting the antiviral action of interferons or down-regulating viral RNA synthesis. J Virol 76: 7114-7124.

38. Kato A, Cortese-Grogan C, Moyer SA, Sugahara F, Sakaguchi T, et al. (2004) Characterization of the amino acid residues of sendai virus C protein that are critically involved in its interferon antagonism and RNA synthesis down-regulation. J Virol 78: 7443-7454.

39. Grogan CC, Moyer SA (2001) Sendai virus wild-type and mutant C proteins show a direct correlation between L polymerase binding and inhibition of viral RNA synthesis. Virology 288: 96-108.

40. Irie T, Yoshida A, Sakaguchi T (2013) Clustered Basic Amino Acids of the Small Sendai Virus C Protein Y1 Are Critical to Its Ran GTPase-Mediated Nuclear Localization. PLoS One 8: e73740.

41. Kato A, Kiyotani K, Kubota T, Yoshida T, Tashiro M, et al. (2007) Importance of the anti-interferon capacity of Sendai virus C protein for pathogenicity in mice. J Virol 81: 3264-3271.

42. Sakaguchi T, Kato A, Sugahara F, Shimazu Y, Inoue M, et al. (2005) AIP1/Alix is a binding partner of Sendai virus C protein and facilitates virus budding. J Virol 79: 8933-8941.

43. Boonyaratanakornkit J, Bartlett E, Schomacker H, Surman S, Akira S, et al. (2011) The C proteins of human parainfluenza virus type 1 limit double-stranded RNA accumulation that would otherwise trigger activation of MDA5 and protein kinase R. J Virol 85: 1495-1506.

44. Van Cleve W, Amaro-Carambot E, Surman SR, Bekisz J, Collins PL, et al. (2006) Attenuating mutations in the P/C gene of human parainfluenza virus type 1 (HPIV1) vaccine candidates abrogate the inhibition of both induction and signaling of type I interferon (IFN) by wild-type HPIV1. Virology 352: 61-73.

45. Bartlett EJ, Cruz AM, Esker J, Castano A, Schomacker H, et al. (2008) Human parainfluenza virus type 1 C proteins are nonessential proteins that inhibit the host interferon and apoptotic responses and are required for efficient replication in nonhuman primates. J Virol 82: 8965-8977.

46. Bartlett EJ, Amaro-Carambot E, Surman SR, Collins PL, Murphy BR, et al. (2006) Introducing point and deletion mutations into the P/C gene of human parainfluenza virus type 1 (HPIV1) by reverse genetics generates attenuated and efficacious vaccine candidates. Vaccine 24: 2674-2684.

47. Schomacker H, Hebner RM, Boonyaratanakornkit J, Surman S, Amaro-Carambot E, et al. (2012) The C proteins of human parainfluenza virus type 1 block IFN signaling by binding and retaining Stat1 in perinuclear aggregates at the late endosome. PLOS ONE 7: e28382.

48. Boonyaratanakornkit JB, Bartlett EJ, Amaro-Carambot E, Collins PL, Murphy BR, et al. (2009) The C proteins of human parainfluenza virus type 1 (HPIV1) control the transcription of a broad array of cellular genes that would otherwise respond to HPIV1 infection. J Virol 83: 1892-1910.

49. Newman JT, Riggs JM, Surman SR, McAuliffe JM, Mulaikal TA, et al. (2004) Generation of recombinant human parainfluenza virus type 1 vaccine candidates by importation of temperature-sensitive and attenuating mutations from heterologous paramyxoviruses. J Virol 78: 2017-2028.

50. Bartlett EJ, Amaro-Carambot E, Surman SR, Newman JT, Collins PL, et al. (2005) Human parainfluenza virus type I (HPIV1) vaccine candidates designed by reverse genetics are attenuated and efficacious in African green monkeys. Vaccine 23: 4631-4646.

51. Durbin AP, McAuliffe JM, Collins PL, Murphy BR (1999) Mutations in the C, D, and V open reading frames of human parainfluenza virus type 3 attenuate replication in rodents and primates. Virology 261: 319-330.

52. Malur AG, Hoffman MA, Banerjee AK (2004) The human parainfluenza virus type 3 (HPIV 3) C protein inhibits viral transcription. Virus Res 99: 199-204.

53. Mao H, Chattopadhyay S, Banerjee AK (2009) N-terminally truncated C protein, CNDelta25, of human parainfluenza virus type 3 is a potent inhibitor of viral replication. Virology 394: 143-148.

54. Mao H, Chattopadhyay S, Banerjee AK (2010) Domain within the C protein of human parainfluenza virus type 3 that regulates interferon signaling. Gene Expr 15: 43-50.

55. Wells G, Addington-Hall M, Malur AG (2012) Mutations within the human parainfluenza virus type 3 (HPIV 3) C protein affect viral replication and host interferon induction. Virus Res 167: 385-390.

56. Malur AG, Wells G, McCoy A, Banerjee AK (2009) Evidence for phosphorylation of human parainfluenza virus type 3 C protein: mutant C proteins exhibit variable inhibitory activities in vitro. Virus Res 144: 180-187.

57. Caignard G, Komarova AV, Bourai M, Mourez T, Jacob Y, et al. (2009) Differential regulation of type I interferon and epidermal growth factor pathways by a human Respirovirus virulence factor. PLoS Pathog 5: e1000587.
