# Supplementary material for: Evolution and Structural Organization of the C Proteins of Paramyxovirinae
Source: PLoS One. 2014 Feb 25;9(2):e90003. doi: 10.1371/journal.pone.0090003 (PMC3934983; doi:10.1371/journal.pone.0090003)
Supplement: File S1 — Multiple sequence alignment of the C proteins of the measles, Nipah, and Sendai groups. (DOC) [file pone.0090003.s003.doc]

**Supporting File S1. Multiple sequence alignment of the C proteins of the measles, Nipah, and Sendai groups.**

The alignments were made using Psi-Coffee (see Methods). The alignment of the measles, Nipah and Sendai group are in pages 1, 2 and 3 respectively.

**Sequence alignment of the C proteins of the measles group**

>Measles

MSKTDWNASGLSRPSPSAHWP-SRKPWQHGQKYQTT----QDRTEPP-AR-KRRQAVR-------------V

SANHASQQLDQLKAVHLASAVR-DLEKAMTTL-KLWESP-Q-EI--SRHQALGYSVIMFMITAVKRLRESKM

LTLSWFNQALMVIAPSQEETMNLKTAMWILANLIPRDMLSLTGDLLPSLWGSGLLMLKLQKEGRSTSS

>Rinderpest

MSTKVWRASKPLGLDLSTPRS-LKKPLQHGSRPQRG---TRLIGCPP--M-RPRQTIR-------------V

PAGHAAQQLDQAKAASLAVTIR-DLEKATAVM-GSWEHS-W-MT--SQCTIPRYSVIMFLITAVKRLRESKM

LTLSWFSQALMMVSDSGEEMKNLRTAMWILANLIPREVLPLTGDLLPLLQQQEPLM---------LKQ

>Canine DV

MSAKGWNASKPSERILLTLRR-FKRSAASETKPATQ----AKRMEPQ-AC-RKRRTLR-------------I

SMNHTSQQKDQTMSAMYLKIIR-DVENAILRL-WRRSGP-L-ER--TSNQDLEYDVIMFMITAVKRLRESKM

LTVSWYLQALSVIEDSREEKEALMIALRILAKIIPKEMLHLTGDILSALNRTEQ------------LM

>Dolphin MV

MSIRDLSVSNLSEKIRPMLSK-LRKPKLSEARPPAK----NQARVIT-RT-TPKKTLL-------------I

STNHALQQLDQKRTACYLVMIQ-DLEHQVTSL-MKESPS-Q-ET--SERRNLQYDVTMFMITAVKRLKESRM

LTCSWFQQAVMMMQNSETEMRALSRAMVNLALLIPEEILPLTGDLLPGLRSRDRLT---------LRL

>Feline MV

MASNLYKRSKTTLRLPKMSIL-PGRFTNPLDKQEHL----QCKEEPLREI-ILRQGVTMTQ-----------

-----CIAKDQVLLFQVLTRISKDLLDSI---I----Q-DYG-I--QRVTSACYSKAMMMKTIIQRLMAGLP

LSKDWMNRIMRTQLLNNQEISVLRECLRLIHLLVPRKLHYLLGDLIYLGQEYQPVP---------LWI

>Salem

MKIEHLSRM-LSRFLQILKSKRINPLENQESHYRTLESITEKTTMPN-SGKTSKKSIYLCK-----------

--RDPSAMSNSASRVEIKTIAH-ELRQEIRKEISKWIQKQQVKS--VVPQDL-HVETQILLTMLSRLAEGEL

LTQDWQRSVLKEIQDSAITCEVMDQLVAMILDLFPRKYHDQVQGIQPI-----PVM---------LGV

>Nariva

MPSRFLQSLRKLTLRR--------QRTNAEEDSQSR----ESPPRPP-PR-TPRPRVRVGVRKN--P--DLV

GVEKETQARKQAEAINLLTMLR-DMEREN---L------RGL-R--GLEGLTEYTTVQFVKTILMRVSEGSP

VTSCWVQQVEEHICQSQREKEALHEAVQWVRAVM--------------------------------QK

>Mossman

MPSKFWQSLKRLRVPS--------RKRSSESDSTYQ----ELQPQPP-QI-PLRPRVKIGVRRN--P--DLV

GVERSNKAQHQVLALDLLNTLK-EMEMEY---P------MEG-P--MAPFRLEYSTLQFVKTILMRVSEGHL

VTSCWARQVELNLCQTQQEIENLHEAISWVKMMMQDN-----------------------------QD

>TPMV

MPLKFWKRLMPPKKKSS--------------ETLTL----LSNQEPL-SM-QDPPLVRSSTRSSIYPPVIKK

GEHRAKTKRNQELAEQLLKELPH-ETTSI--------A-NLV-Q--RNNRDLDYNLEQLVRTLLQMEKEGTH

VTESLINTLMETDTLTPKEQALIWPAYNLVRQMMHHA-----------------AL---------HHI

**Sequence alignment of the C proteins of the Nipah group**

>Nipah

--------MMASILLTLFRRTKKKYRRHTDDQAFNNPASKTEQKHGRIFCSAPVENLNKLRGECLRMMEVLK

EEAWR----IYPVLLPQMELLEKECRT----PVTGQKVQ-MTYNWTQWLQTLYTMIME-ENVPDMDLLQALR

EGGVITHQEQTMGMYVLYLMQRCCPMLPKLQFLKKIG----------------KLI

>Hendra

--------MMASILLTLFRRTKKKYKRHTDDQASNNQVPKTGQEHGRTSCRAPVENMNRLRGECLRMMEVLK

EEMWR----IYPVLLPQMELLDKECQT----PELGQKTQ-MTYNWTQWLQTLYTMIME-ENVPDMDLLQALR

EGGVITCQEHTMGMYVLYLIQRCCPMLPKLQFLKKLG----------------KLI

>Cedar

--------M-ASLLSILYRKIRKNYSILTEDPPSESHPQVSGLKSGRNLFERSLLDLNKFKGE----DLRLR

SQAIMEIEAILPILIREAESQDNSKKG----IKNGGH-KIQNYNWTQWLYTISSMTRE-GRIPTMENMTAAL

KNGIISEKEHDRISTIISLLMNYCP---AYNHLLR-TMSSRMKVHQCQICMLQEIN

>Bat PMV

--------M-ASKLLNSFRKIRMRSKRPMEDPKFKSQEPEKEQQPGSSLFGVRIQDPKEIEWLEWINKIQLR

DEMTM----ADPILLPEMERLEKAVPI----ISHGPAGE-MIYSWVQWYKTLRSMIME-LNYPTLEGLNQLL

DAGVLTRAEHQIGKEVLRVISTICP---TYQVVGKIL----------------ETI

>Beilong

MSSSSLSRM-ESRLSSFYSRVRKTFRRRTAEVPSRSRRQEPESKVGSPAIPIMITPVIKIREA----RELKR

EQTRA----RARELLQLMEDMEINPQITGVTPKTSTKGQINRYGML---RIMMGIVAELGEVPQVDF-PRLE

KEGILSQQEIKNLRDT-------IP---TVQLMLE-S----------------IIR

>Jvirus

--------M-ESKLPNFFRRIRRTFRRRTGEVQLDYQPPKKESQPGKPLQRLPMESRSSWVEE----TEKIR

EENKL----KAKTILVAMDMVEKRYPPAQILQLYSCRDLITKYGMV---RMLLLIVAEKGEIPGINM-GGLI

QARLLTQEEVTNLMEA-------VP---IVRLMLE-T----------------LGK

**Sequence alignment of the C proteins of the Sendai group**

>Sendai

MAS---ATL-PAWIKMPSFLKKILKLR-GRRQEDESRSRMLSDSSTQSYQVNQLTSEGTEAGSTIPSTPSKG

QALPTESKVRAREKSRHRRPKI---IDQVRRVESLGEQASQRQKHMLETLINKIY-TGPLGEELVQTLYL--

RIWAMEETPESLKILQMREDIRDQVLKMKTERWLRTLIRGEKTKLKDFQKRYEEVHPYLMKEKVEQIIMEEA

WSLAAHIVQE---------------------------------------------

>hPIV1

MDTSASKTLLPEWIRMPSFLRGILKPK-ERHHENKNHSQMSSDSLTSSYPTSPQKLEKTEAGSMVSSTTQKK

TSHHAKPTITTKTEQSQRRPKI---IDQVRGVESLGEQVSQKQRHMLESLINKVY-TGPLGEELVQTLYL--

RIWAMKETPESTKILQMREDIRDQYLRMKTERWLRTLIRGKKTKLRDFQKRYEEVHPYLMMERVEQIIMEEA

WKLAAHIVQE---------------------------------------------

>pPIV1

---------------MPSFLKNLWRNK-RRDTQQPAHSPVQSDSSTSSLPVSPQTLEKIENTYVSPSQLGEN

QMQARFLRVQSVKKTQEQKGKI---MDKVKRVEFLGSQTSLKQKFLLERLIAQIH-HGGLGEEVVQTLYL--

RIWAMDPTPMATKLLEMEEEIRDKVLKLKLERWIRVLIRGEKTKLRDFQKRYEEVHPYLMTEKVEEIIMEEA

WSLSAHIIQE---------------------------------------------

>bPIV3

---------------MLK---TIKSWILGKRDQE-TS-HLTSHRPSTSLNSYSAPTPKRTRQTAMKSTQGT-

-RDSARQSTNLNPKQQKQAKKI---VDQLTKIDSLGHHTNVPQRQRIEMLIRRLY-REEIGEEAAQIVEL--

RLWSLEESPEAAQILTMEPKSRKVLITMKLERWIRTLLRGKCDNLKMFQSRYQEVMPFLQQNKMETVMMEEA

WNLSVHLIQDIPA------------------------------------------

>hPIV3

---------------MLK---TIKSWILGKRNQE-IN-QLISPRPSISLNSYLAPTPKKTYRKTTQSTQEP-

-SNSVPPSINQKSNQQKQVRKI---VDQLTKIDSLGHHTNVQQKQKIELLIRKLY-REDLGEEAAQIVEL--

RLWSLEESPEASQILKMEPRTRRILISMKLERWIRTLLRGKCDNLQMFQARYQEVMSYLQQNKVETVIMEEA

WNLSVHLIQDQ--------------------------------------------

>PacificSalmon

---------------MAGLLARINKWR-GKKQADTPMRESLY--STQDFPTFSPSSET----SIPPPEPP--

--KRRNLTVVPMTPRAPTPPQVLTGPGTMLVVHPIGGYDPK-AKTLSPSGILAAYIATIQGARSIAVLTRDL

QDLLLRSPEEGIFIRELLESTR--YLLMRVEMWISLLTRKKRPTYQWLNNRMEQMKRVTLNKPVEDLLLDQA

WVVVVPWLDGEGRQILPVRQKSLRDMTTLRGGQRMQHSQSFSPASRVEQTAYVAQ

>AtlanticSalmon

---------------MSSILDKIKRWQ-NKRATQTSSSMMSS--LTKVSTSSGVSSEE----MPKLPQPK--

--KHRDTRVVKMIPVPLKRPQLPPGEGTVLVVHPIGKCDPR-EKRMSTSGIIVAYIATDSGANSIRALVKSL

EEVKMETPEDSIFVDQMLCSTR--VLLIRIEHWVELLTRGRRPTWNLLISRMALMKRVSQGKPVEELLLDEA

WKVVLPWIEGEVRASLPAKQISEKGQTTPTPGQT----------------QERLL
