# Supplementary material for: Evolution and Structural Organization of the C Proteins of Paramyxovirinae
Source: PLoS One. 2014 Feb 25;9(2):e90003. doi: 10.1371/journal.pone.0090003 (PMC3934983; doi:10.1371/journal.pone.0090003)
Supplement: File S2 — Multiple sequence alignment of the P/C genes of the measles and Nipah groups, based on an alignment of the C proteins (DOC) [file pone.0090003.s004.doc]

**Supporting File S2. Multiple sequence alignment of the P/C genes of the measles and Nipah groups, based on an alignment of the C proteins**

The nucleotide sequences below are exactly those of the C coding sequences. Their alignment was derived, using TranslatorX, from an alignment of the C proteins (see Methods and Figure 8). They can be converted back into the sequences of the C proteins by translating them directly (i.e. in the +0 frame). They can be converted into the sequences of the N-terminal part of the P proteins (PNT) by translating them into the +2 frame (the P frame). For instance, translation of the measles virus C coding sequence below into the +2 frame yields the sequence of aa 9-193 of measles virus PNT. The alignment of the region of PNT presented in Figure 9 is derived from a larger alignment of PNT obtained in this way.

>Measles|9626945:1829-2389_Measles_virus,_complete_genome/1-558

---------------ATGTCAAAAACGGACTGGAATGCATCCGGGCTCTCAAGGCCGAGCCCATCGGCTCAC

TGGCCGTCGAGGAAGCCATGGCAGCATGGTCAGAAA------TATCAGACAACCCAGGACAGGACCGAGCCA

CCTGCAAGGAAG------AGGAGGCAGGCAGTTCGGGTCTCAGCAAACCATGCCTCTCAGCAATTGGATCAA

CTGAAGGCGGTGCACCTCGCATCCGCGGTCAGGGATCTGGAGAAAGCGATGACGACGCTGAAACTT---TGG

GAATCCCCTCAAGAAATCTCCAGGCATCAAGCACTGGGT------------TACAGTGTTATCATGTTTATG

ATCACAGCGGTGAAGCGGTTAAGGGAATCCAAGATGCTGACTCTATCATGGTTCAATCAGGCCTTGATGGTG

ATAGCACCCTCTCAGGAGGAGACGATGAATCTGAAAACAGCGATGTGGATATTGGCGAACCTGATACCGAGG

GATATG------------CTATCACTGACCGGGGATCTGCTCCCATCTCTATGGGGTTCAGGGCTTCTGATG

TTGAAACTGCAGAAGGAGGGGAGATCCACGAGCTCC

>Rinderpest|56410431:1829-2362Rinderpest_virus_KabeteO,completegenome/1-534

---------------ATGTCAACAAAGGCCTGGAATGCATCAAGGCTCTCAGGGCCCGACCCCTCGACCCCC

TGGTCGTTGAAGAAGCCCTTGCAGCATGGGTCGAGA------CCTCCGAAGGGCAAACGCTTGACCGTATGT

CCTCCGACGAGG------CCGAAGCAGACCATCAGGATATCAGCAAGCCATGCTTCCCAGCAGCTGGACCAG

GCAAAAGCAGCATGTCTCGCTGTCACGATCAGGGACTTGGAGGAAGCAACAGCTGTGATGAGGAGC---TGG

GAGCATTCATTGGTGACTCCTCAATGCATAGCACCGAGG------------TACAGCATTATCATGTTTATG

ATCACAGCGGTGAAAAGGTTGAGGGAGTCGAAGATGCTGACTCTATCCTGGTTCAATCAGGCGCTGATGATG

GTGTCGAAGTCTGGGGAGGAGATGAGGAATCTGAGAACAGCGATGTGGATTCTGGCGAACCTGATCCCGAGG

GAAGTG------------CTCCCGCTGACTGGGGATCTTCTCCCATCTCTCCAGCAACAAGAGCCTCCGATG

TTGAAACAGTAG------------------------

>CanineDistemper|9630645:1823-2347_Canine_distemper_virus,_complete_genome/1-525

---------------ATGTCAGCAAAGGGCTGGAATGCCTCAAAGCCCTCAGAGAGAATCCTCCTGACATTG

AGGAGATTCAAGAGGTCAGCAGCCTCAGAGACCAAA------CCTGCAACCCAGGCCAAGAGAATGGAACCA

CAGGCATGCAGG------AAGAGGAGGACTCTCAGAATCTCGATGAATCACACGAGCCAACAAAAGGATCAA

ACTATGTCGGCCATGTACCTCAAAATAATCCGGGATGTGGAGAACGCAATA------CTGCGCTTG---TGG

AGGCGGAGCGGCCCCCTAGAGAGGACATCCAACCAGGACCTG------GAATACGATGTGATCATGTTTATG

ATCACAGCGGTGAAGAGGTTAAGGGAATCGAAGATGCTGACAGTCTCGTGGTACCTGCAGGCACTGTCGGTA

ATCGAGGATTCGAGAGAGGAGAAGGAAGCCTTGATGATAGCACTGAGGATTCTGGCGAAGATTATTCCGAAG

GAAATG------------CTTCATCTAACTGGGGATATTCTTTCGGCCTTAAACCGGACAGAGCAGCTGATG

TGA---------------------------------

>DolphinMorbilli|38707562|Dolphin_morbillivirus,_complete_genome/1-534

---------------ATGTCAATAAGGGACTTGAGTGTCTCAAATCTCTCAGAGAAAATCCGCCCGATGCTG

TCGAAATTAAGGAAGCCCAAATTATCCGAAGCAAGG------CCGCCTGCGAAGAATCAAGCGAGAGTCATC

ACCAGGACAACT------CCGAAAAAGACACTCTTGATTTCGACGAATCATGCTCTTCAGCAATTAGACCAG

AAACGTACCGCATGTTACTTGGTGATGATACAGGATTTAGAGCACCAGGTT------ACATCCCTA---ATG

AAGGAGAGCCCGAGCCAGGAGACATCGGAAAGGAGGAACCTG------CAGTACGATGTTACCATGTTTATG

ATCACGGCGGTCAAGCGGTTGAAGGAGTCAAGGATGCTGACCTGCTCGTGGTTCCAACAGGCAGTGATGATG

ATGCAGAATTCAGAGACGGAGATGAGAGCTCTCTCGAGAGCGATGGTGAATCTGGCACTGTTGATACCAGAG

GAAATT------------CTTCCTCTAACAGGGGATCTGCTCCCAGGATTAAGGTCGAGAGATCGTCTGACG

TTGAGACTATAA------------------------

>FelineMorbilli|JQ411014_1|Feline_morbillivirus_761U,_complete_genome/1-513

---------------------------------ATGGCCTCGAATCTTTACAAGAGATCAAAAACAACCCTC

CGTCTTCCCAAGATGTCAATCTTGCCAGGG---AGA------TTTACGAATCCATTAGACAAACAGGAACAT

CTTCAGTGCAAGGAGGAGCCATTGCGGGAGATAATATTACGTCAGGGGGTAACAATGACTCAATGTATAGCC

AAGGACCAAGTCCTCCTATTTCAAGTGTTAACAAGAATATCGAAGGACCTA------CTGGATTCG---ATC

ATTCAGGACTATGGGATCCAGAGGGTAACCTCTGCATGC------------TATTCGAAAGCGATGATGATG

AAAACCATTATTCAGAGATTAATGGCCGGTCTTCCGCTATCGAAGGACTGGATGAACAGGATAATGAGAACT

CAATTATTAAACAACCAGGAAATCAGTGTACTGAGGGAGTGTCTAAGACTGATTCATCTCTTAGTTCCCAGG

AAACTA------------CACTATCTGTTGGGGGATCTGATATACCTGGGGCAGGAATATCAACCTGTGCCT

CTTTGGATATAA------------------------

>Salem|AF237881_1|:1820-2359|Salem_virus_N,_V,_and_C_protein_mRNA/1-540

---------ATGAAAATAGAACACTTGTCAAGAATGCTCTCAAGGTTCTTGCAGATCTTAAAAAGCAAGAGA

ATAAACCCATTGGAGAACCAAGAATCACATTACAGAACCTTAGAGAGCATTACGGAAAAAACGACTATGCCA

AATTCGGGGAAG---ACCTCCAAGAAGTCCATTTATCTCTGCAAAAGAGACCCCAGTGCAATGTCGAACTCC

GCCAGCCGGGTGGAGATCAAGACAATAGCCCACGAATTAAGACAGGAAATCAGGAAGGAAATCTCCAAGTGG

ATACAGAAACAACAGGTGAAGTCAGTTGTACCGCAGGATCTT---------CATGTGGAGACTCAGATTCTC

CTCACGATGCTGAGCAGGCTAGCAGAAGGGGAGTTGCTGACGCAGGACTGGCAGCGTTCTGTACTGAAGGAA

ATTCAGGATTCAGCAATCACATGCGAGGTCATGGACCAGTTAGTGGCGATGATTTTGGACTTGTTCCCGCGA

AAGTAC------------CACGATCAAGTGCAAGGGATCCAACCTATT---------------CCAGTGATG

TTAGGGGTGTGA------------------------

>Nariva|387935515:1783-2232_Nariva_virus,_complete_genome/1-450

------------------------ATGCCCTCCAGGTTCTTGCAGTCGTTAAGGAAACTGACTCTCCGACGA

CAGAGAACCAATGCGGAAGAAGACTCTCAGTCCAGGGAGTCACCACCCAGACCTCCACCACGGACCCCGAGA

CCAAGGGTGAGA------GTGGGAGTGAGGAAGAATCCGGATCTGGTTGGGGTGGAGAAGGAAACTCAAGCC

AGGAAGCAAGCGGAAGCAATCAACTTGTTG---ACAATGCTGAGAGATATG------GAGAGGGAG------

AATCTAAGGGGC------CTCAGGGGCCTGGAGGGTTTGACG------GAGTACACGACAGTCCAGTTCGTC

AAGACCATCCTGATGAGGGTATCAGAAGGGAGCCCTGTTACGTCTTGTTGGGTGCAGCAAGTGGAGGAGCAT

ATCTGTCAGAGTCAGAGGGAGAAGGAAGCCCTACACGAGGCAGTCCAGTGGGTGAGAGCGGTG---------

------------------ATGCAAAAGTGA------------------------------------------

------------------------------------

>Mossman|41057593:1933-2391_Mossman_virus,_complete_genome/1-459

------------------------ATGCCCTCCAAATTCTGGCAGTCGCTAAAGAGACTCAGAGTCCCAAGT

CGGAAGAGGAGCTCAGAGAGCGACTCGACATATCAGGAGTTACAACCACAGCCTCCACAGATACCGTTGAGG

CCGAGAGTCAAG------ATAGGAGTGAGAAGGAACCCGGATCTGGTCGGAGTGGAGAGGAGCAACAAAGCT

CAGCATCAAGTTCTAGCGTTGGATTTACTC---AATACGCTGAAGGAGATG------GAGATGGAG---TAT

CCGATGGAGGGGCCAATGGCTCCATTT---------CGACTA------GAGTATTCTACTCTACAGTTTGTG

AAAACAATCCTGATGAGAGTCTCGGAAGGTCATCTTGTTACGTCATGCTGGGCAAGGCAGGTGGAATTGAAC

CTGTGCCAAACACAGCAGGAGATAGAGAATCTACACGAAGCAATCAGCTGGGTGAAGATGATG---------

------------------ATGCAGGACAATCAGGATTAA---------------------------------

------------------------------------

>Tupaia|9634968:2161-2622_Tupaia_paramyxovirus,_complete_genome/1-462

---------------------------------ATGCCTCTAAAGTTTTGGAAGCGATTGATGCCGCCAAAG

AAGAAGAGCTCAGAAACCTTAACTCTCTTGTCCAAC---------CAAGAGCCCCTCTCAATGCAGGATCCA

CCCCTGGTGAGATCATCAACGAGATCAAGCATCTATCCACCCGTGATCAAGAAGGGGGAACATCGAGCAAAG

ACGAAGAGGAATCAG------GAGCTGGCAGAACAGTTGCTGAAGGAGCTG------CCACACGAG---ACC

ACAAGTATAGCAAATCTCGTCCAAAGAAACAACCGAGATCTG------GATTACAATCTGGAGCAGCTGGTA

AGAACCCTACTCCAGATGGAGAAGGAGGGGACACATGTAACAGAGAGCTTGATCAACACTCTGATGGAGACG

GACACTCTAACACCGAAGGAGCAAGCTCTGATCTGGCCAGCATACAACCTTGTCAGACAGATGATGCACCAT

GCAGCT------------CTACATCATATTTAG---------------------------------------

------------------------------------

>Nipah|13559808:2428-2928_Nipah_virus,_complete_genome/1-501

---------------------ATGATGGCCTCAATATTATTGACTTTATTCAGAAGAACCAAAAAGAAATAC

AGAAGACATACGGACGATCAAGTATTCAACAACCCA------GCATCAAAGATCAAACAAAAGCCTGGGAAG

ATTTTCTGCAGT------GCACCAGTGGAGAATCTGAACAAGTTGAGGGGGGAATGTCTAAGGATGATGGAG

ATGTTGAAAGAAGAAACTTGGAGGATCTATCCAGTACTTCTCCCACAGATG------GAACTATTG---GAA

AGAGAGTGTCGAACACCCGTGACTGGGCAGAAGGTTCAGATG------ACATACAACTGGACCCAGTGGTTA

CAGACGTTGTATACCATGATCATGGAGGAGAATGTACCGGATATGGATTTACTTCAAGCCCTGAGAGAGGGT

GGAGTGATTACACATCAGGAGCAAACAATGGGAATGTATGTCTTGTATCTGATGCAAAGATGCTGTCCTATG

CTCCCGAAATTGCAGTTTCTAAAGAAGATCGGGAAACTGATCTAG---------------------------

------------------------------------

>Hendra|29468603:2410-2910_Hendra_virus,_complete_genome/1-501

---------------------ATGATGGCCTCGATATTATTGACTTTATTCAGAAGAACCAAAAAGAAATAC

AAAAGACATACGGACGATCAAGCATCCAACAACCAA------GTACCAAAGACAGGACAAGAGCATGGGAGG

ACTTCTTGCAGA------GCACCAGTGGAGAACATGAACAGGCTGAGGGGGGAATGCCTAAGAATGATGGAG

GTACTGAAGGAAGAAATGTGGAGGATCTATCCAGTGTTACTTCCTCAGATG------GAACTATTG---GAC

AAAGAGTGTCAAACACCCGAGCTTGGGCAGAAGACCCAGATG------ACATACAACTGGACCCAATGGTTA

CAGACGTTGTATACCATGATCATGGAGGAGAATGTACCGGACATGGACCTTCTTCAAGCCCTGAGAGAGGGT

GGAGTTATCACATGTCAGGAACACACGATGGGAATGTACGTGCTGTACCTGATACAAAGGTGTTGCCCAATG

CTCCCAAAACTACAGTTCCTGAAGAAGTTAGGGAAATTGATTTAA---------------------------

------------------------------------

>Cedar|JQ001776_1|:2137-2670Cedar_virus_isolate_CG1a,_complete_genome/1-534

------------------------ATGGCCTCTCTACTATCAATTTTATACAGGAAAATAAGGAAAAATTAC

AGCATTCTTACGGAAGATCCTCCATCAGAGAGCCAC------CCACAAGTGTCAGGGTTGAAGAGTGGGAGA

AATTTATTCGAA------AGATCGCTTCTGGACCTGAACAAGTTCAAGGGGGAGGATCTGAGACTGAGATCA

CAGGCGATAATGGAGATAGAGGCAATTTTACCAATCCTGATCAGGGAGGCG------GAGTCACAG---GAC

AATTCGAAGAAAGGTATCAAAAATGGGGGTCACAAGATTCAG------AATTACAACTGGACCCAATGGTTG

TACACGATTTCTTCTATGACGAGAGAAGGGAGAATCCCGACAATGGAAAATATGACCGCAGCTCTAAAAAAC

GGGATAATATCAGAGAAGGAACACGACAGGATAAGTACAATAATCAGTCTACTGATGAATTACTGTCCTGCC

TACAACCATCTT------CTAAGAACGATGTCATCAAGAATGAAAGTACATCAGTGTCAAATTTGCATGTTA

CAGGAAATAAACTGA---------------------

>BatHenipavirus|HQ660129_1|:2078‑2566_BatHenipavirus|Bat_Paramyxovirus_Eid_hel/GH-M74a/GHA/2009,_complete_genome/1-489

------------------------ATGGCATCAAAATTATTGAATTCATTCAGAAAAATAAGGATGAGATCC

AAAAGACCTATGGAAGATCCCAAATTCAAGAGCCAA------GAACCAGAGAAAGAGCAGCAGCCTGGGAGC

TCTTTATTCGGG------GTGAGAATCCAAGACCCGAAGGAGATAGAATGGCTGGAGTGGATCAACAAGATC

CAACTGAGAGACGAGATGACGATGGCAGACCCAATACTCTTGCCAGAGATG------GAGAGATTG---GAG

AAAGCGGTCCCAATTATATCTCATGGTCCAGCGGGGGAGATG------ATATACAGTTGGGTCCAATGGTAC

AAGACTTTGAGATCGATGATAATGGAATTAAACTACCCAACTCTAGAGGGTCTAAATCAGCTCCTAGATGCA

GGAGTGTTGACAAGAGCAGAGCATCAGATTGGGAAAGAAGTCCTGCGAGTAATATCGACCATTTGTCCAACC

------------------TATCAGGTGGTGGGAAAGATCTTGGAAACTATATAA------------------

------------------------------------

>Beilong|89888070:1867-2349_Beilong_virus,_complete_genome/1-483

ATGTCCTCCAGCAGCTTGTCAAGGATGGAATCAAGACTATCGAGCTTTTACAGCAGAGTCCGGAAGACTTTC

AGAAGACGTACGGCAGAAGTGCCATCCAGGAGCCGT------CGACAAGAGCCAGAATCCAAAGTTGGGAGT

CCCGCAATCCCG------ATCATGATTACACCGGTAATAAAAATCAGAGAAGCGAGGGAGCTAAAGAGAGAG

CAAACAAGAGCGAGAGCGCGGGAACTGCTT---CAGCTGATGGAGGACATG------GAGATAAAC---CCT

CAAATAACGGGGGTGACTCCCAAAACGAGTACCAAGGGTCAGATCAACAGGTATGGGATGCTGCGTATAATG

ATGGGAATAGTGGCGGAGCTTGGGGAGGTCCCACAGGTGGAC---------TTCCCACGGCTGGAGAAAGAG

GGTATCCTATCACAACAGGAAATCAAGAATTTGAGGGATACCATCCCGACGGTCCAGTTGATG---------

------------------CTCGAGAGTATAATCAGATAA---------------------------------

------------------------------------

>Jvirus|77124337:1873-2331_J-virus,_complete_genome/1-459

------------------------ATGGAATCAAAACTGCCCAATTTTTTCAGAAGAATCAGGAGAACATTC

AGAAGACGTACGGGAGAAGTGCAATTGGACTACCAA------CCACCAAAGAAAGAATCTCAGCCTGGGAAG

CCGTTGCAGAGA------CTCCCTATGGAGAGCAGATCCAGCTGGGTGGAGGAAACGGAGAAGATCAGAGAG

GAGAACAAGCTGAAGGCCAAGACAATCCTG---GTGGCCATGGACATGGTG------GAGAAGAGA---TAC

CCACCGGCGCAAATCCTTCAGCTTTACAGTTGCAGGGATCTTATAACCAAGTATGGGATGGTCCGAATGTTG

CTACTTATAGTGGCGGAGAAGGGGGAGATACCGGGAATCAAC---------ATGGGAGGACTGATTCAGGCT

CGGCTGCTGACACAGGAGGAAGTAACCAATCTGATGGAAGCAGTACCTATAGTACGGTTGATG---------

------------------CTGGAGACCTTAGGCAAATGA---------------------------------

------------------------------------
